# Phage Therapy in Combating Multidrug-Resistant Gram-Negative Pathogens: A Scoping Review

**DOI:** 10.3390/ph19050727

**Published:** 2026-05-03

**Authors:** Asif Sukri, Bruno Silvester Lopes, Alfizah Hanafiah

**Affiliations:** 1 Department of Biological Sciences and Biotechnology, Faculty of Science and Technology, Universiti Kebangsaan Malaysia, Bangi 43600, Selangor, Malaysia; mohdasifsukri@ukm.edu.my; 2 School of Health and Life Sciences, Teesside University, Middlesbrough TS1 3BA, UK; 3 National Horizons Centre, Teesside University, Darlington DL1 1HG, UK; 4 Department of Medical Microbiology & Immunology, Faculty of Medicine, Universiti Kebangsaan Malaysia, Cheras, Kuala Lumpur 56000, Malaysia

**Keywords:** phage therapy, *Pseudomonas aeruginosa*, *Acinetobacter baumannii*, *Klebsiella pneumoniae*, *Helicobacter pylori*, multidrug resistance

## Abstract

**Background:** The emergence of multidrug-resistant (MDR) Gram-negative pathogens, namely *Klebsiella pneumoniae*, *Pseudomonas aeruginosa*, *Acinetobacter baumannii* and *Helicobacter pylori*, necessitates urgent therapeutic alternatives. This scoping review aimed to summarize the current evidence on the efficacy of lytic bacteriophages against these critical MDR pathogens, and to identify existing research gaps and implementation challenges. **Methods:** The literature search was conducted by searching PubMed, Web of Science, and Scopus AI for studies published from 2015 to 2025. The inclusion criteria focused on experimental and human studies evaluating phage therapy against MDR, extensively drug-resistant (XDR), or pan-drug-resistant (PDR) strains in the four target species. A total of 172 articles were included. **Results:** A number of studies showed an increasing trend (2015–2025), focusing mainly on *K. pneumoniae* (n = 65), *P. aeruginosa* (n = 55), and *A. baumannii* (n = 48). No eligible studies for MDR *H. pylori* were found. All 172 studies confirmed lytic activity, with phage cocktails showing superior antibacterial activity than single phages in four studies. Phages also demonstrated antibiofilm activity (n = 44). Most animal studies reported successful bacterial reduction in animals treated with phages, and 87.5% of 23 human case studies reported patient improvement or infection clearance. However, heterogeneity in the types of animal models used and in dosage and administration routes in human studies was notable. **Conclusions:** Lytic bacteriophages exhibit strong potential as a new therapeutic option. Key challenges include the lack of data for MDR *H. pylori*, heterogeneity in animal models, and a paucity of large-scale human clinical trials. Future research must prioritize standardization, mechanistic studies, and conducting robust human trials to enable clinical translation and regulatory acceptance.

## 1. Introduction

The emergence of multidrug-resistant bacteria has complicated treatment strategies for treating infections caused by clinically important bacteria [1]. Recently, the World Health Organization published a list of clinically important bacteria that require urgent attention in the research and development of new therapies beyond antibiotics. The list includes Gram-negative pathogens with critical priority in the research and discovery of new therapies, namely carbapenem-resistant *Acinetobacter baumannii* and *Klebsiella pneumoniae*. In addition, carbapenem-resistant *Pseudomonas aeruginosa* was also included as a high-priority pathogen. The pathogens were included in the priority list as they cause high mortality annually, are easily transmitted in a clinical setting, and are difficult to treat when they are resistant to last-resort antibiotic options [2]. Another clinically important Gram-negative bacterium is *Helicobacter pylori*. Although it is no longer included in the revised list of WHO high-priority pathogens, *H. pylori* is still regarded as a clinically important pathogen, as infection with this bacterium causes gastroduodenal diseases including gastric cancer and peptic ulcer [3]. Gastric cancer is one of the most common cancers in the world that results in almost a million deaths annually [4]. Thus, eradication of *H. pylori* is pertinent to prevent development of gastric cancer. However, the rise in antibiotic-resistant *H. pylori* has complicated eradication strategy [5].

Bacteriophages have been explored as alternative in combating antibiotic-resistant bacteria [6]. They are attractive option to be used in treatment of bacterial infection because they are safe, highly specific to targeted bacterial species, and are abundant in environment where they can be easily isolated [7]. While bacteriophages have been demonstrated to be good therapeutic option for bacterial infection in vitro and in vivo, studies in humans are lacking [8]. In addition, studies have focused on antibiotic-susceptible bacteria without taking into account the antibiotic resistance profile of bacteria used in the experiment [9]. Studies published are also heterogenous in terms of experimental design and approaches used [10]. Bacteria can also develop resistance to phages with mechanism not fully understood [11]. Thus, it is necessary to understand studies published to date on phages to identify gaps and highlight strategies for overcoming any challenges.

This scoping review aimed to summarize current evidence on efficacy of lytic bacteriophages against clinically important bacteria and to identify gaps in the studies published.

## 2. Materials and Methods

This scoping review was developed using the Arksey and O’Malley (2005) framework [12], and it was carried out in accordance with the Preferred Reporting Items for Systematic Reviews and Meta-Analysis Extension for Scoping Reviews [12]. The following steps were implemented: (1) defining the research question; (2) gathering the relevant studies; (3) selecting the studies; (4) extracting the data; (5) reporting the results.

### 2.1. Defining the Research Question

The research questions were as follows: What is the current evidence on the use of bacteriophage therapy for infections caused by MDR *K. pneumoniae*, *A. baumannii*, *P. aeruginosa* and *H. pylori*? We further refined the parameters to further sub-questions: (a) Which MDR Gram-negative pathogens are most studied? (b) What types of phage therapy approaches have been used against these MDR pathogens? (c) What clinical outcomes or experimental results are documented for these infections? (d) What are the limitations, knowledge gaps and barriers to translating page therapy into routine treatment for these pathogens?

### 2.2. Inclusion and Exclusion Criteria

Inclusion and exclusion criteria were defined according to the PCC framework (Population-Concept-Context) recommended by the Joanna Briggs Institute (JBI) [13] as follows:

Population (P): Multidrug-resistant Gram-negative bacterial pathogens—specifically *Klebsiella pneumoniae*, *Acinetobacter baumannii*, *Pseudomonas aeruginosa* and *Helicobacter pylori*, Concept (C): Use or evaluation of phage therapy (natural or phage cocktails or phage-antibiotic combinations) and Context (C): in vitro, in vivo (animal), and clinical or therapeutic applications across global settings.

Inclusion criteria for this study include: (1) Study focus: Studies investigating bacteriophage therapy or phage-based treatments targeting MDR *K. pneumoniae*, *A. baumannii*, *P. aeruginosa* or *H. pylori*. Only studies evaluating naturally occurring lytic bacteriophages for therapeutic purposes were included. (2) Bacterial resistance: Studies that explicitly mention antibiotic resistance, multidrug resistance (MDR), extensively drug-resistant (XDR), or pan-drug-resistant (PDR) strains, (3) Type of study: Experimental studies (in vitro, animal models, biofilm studies, infection models) and human studies (clinical trials, case reports, case series, and observational studies), (4) Phage type: Natural phages; phage cocktails; phage-antibiotic synergy studies, (5) Outcomes reported: Any outcomes related to antibacterial efficacy, resistance suppression, infection clearance, biofilm degradation, safety, or treatment response, (6) Publication type: Peer-reviewed journal articles, (7) Language: English and (8) Publication period:2015–2025.

Exclusion criteria for this study include: (1) Bacterial species: Studies not involving the four target species (*K. pneumoniae*, *A. baumannii*, *P. aeruginosa*, *H. pylori*). Studies on veterinary isolates. (2) Non-MDR strains: Studies using antibiotic-susceptible or unspecified strains without reference to resistance profiles. (3) Non-phage interventions: Studies on antibiotics, vaccines, probiotics, or non-phage antimicrobial agents (unless combined with phage therapy) and phage resistance. (4) Non-therapeutic studies: Studies focused only on phage isolation, taxonomy, or genome sequencing without testing therapeutic potential. Studies focusing exclusively on the molecular or mechanistic aspects of bacteriophages (e.g., phage biology, replication mechanisms, or host–phage interactions) without evaluating therapeutic applications. Studies investigating lysogenic (temperate) bacteriophages or phages with lysogenic life cycles. Studies involving genetically modified, engineered, or synthetic bacteriophages designed for therapeutic use. Studies focusing on temperate bacteriophages, prophage induction, or their roles in bacterial physiology, biofilm formation, or resistance modulation without direct therapeutic application were excluded. (5) Reviews, editorials, short communication, or commentaries: Excluded from data synthesis (may be used for background context only). (6) Language: Non-English papers. (7) Unavailable free full text: Abstract-only papers or inaccessible full texts including conference papers, proceedings, and theses/dissertations. (8) Grey literature such as conference papers, theses and reports were excluded from this scoping review due to lack of peer review process.

### 2.3. Identifying Relevant Studies

A literature search was performed via three electronic databases (PubMed, Scopus, and Web of Science) in October 2025 using the following search string: phage therapy AND (multidrug-resistant OR antimicrobial resistance) AND (*Klebsiella pneumoniae* OR *Pseudomonas aeruginosa* OR *Acinetobacter baumannii* OR *Helicobacter pylori*). Articles were selected and screened based on the inclusion and exclusion criteria stated above. First, all retrieved articles were screened by title, and those deemed irrelevant were excluded. Subsequently, the remaining articles underwent abstract screening, with non-relevant studies excluded at this stage. Articles that passed both title and abstract screening were then assessed for full-text eligibility. The inclusion and exclusion process were conducted independently by two researchers, and any disagreements were resolved through consultation with a third researcher. Backward citation searching was performed by screening the reference lists of all included studies to identify additional relevant articles not captured through database searching. All potentially eligible studies identified through this process were assessed against the predefined inclusion and exclusion criteria.

### 2.4. Study Selection

Zotero software (Version 9) was used to compile the literature. The search results from the three electronic databases were downloaded. The software was also used to identify and eliminate duplicate entries, and the list was then reviewed manually.

## 3. Results

### 3.1. Screening of Articles

**Figure 1:**
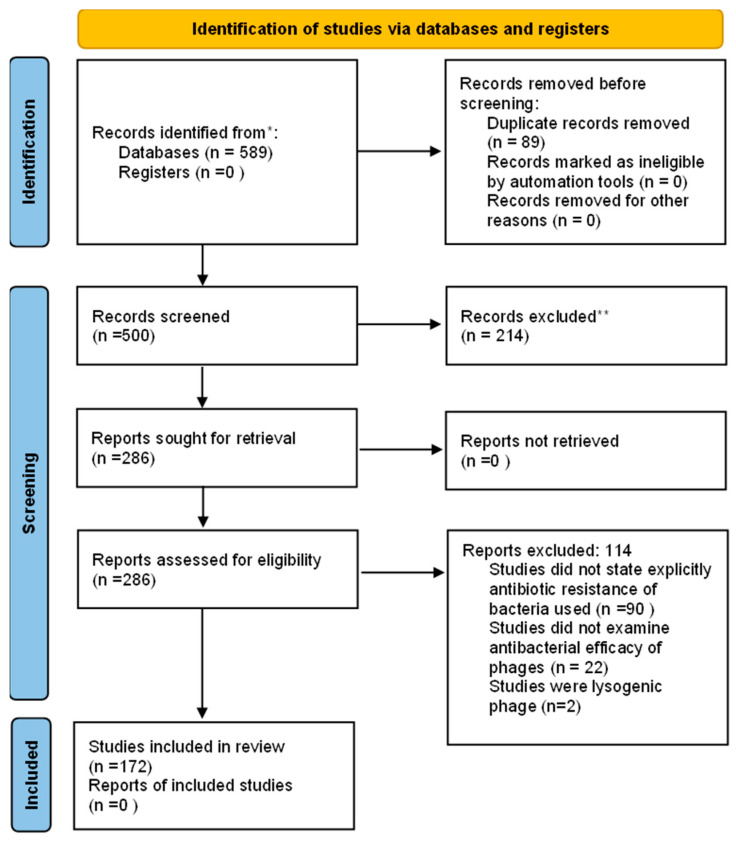
Screening of articles using PRISMA guideline. * Consider, if feasible to do so, reporting the number of records identified from each database or register searched (rather than the total number across all databases/registers). ** If automation tools were used, indicate how many records were excluded by a human and how many were excluded by automation tools.

In this scoping review, the literature search was conducted using three databases, namely PubMed, Web of Science, and Scopus AI. Overall, 582 articles were identified using keywords provided in Section 2. Of 582 articles, 399 articles were obtained from PubMed, 11 articles from Scopus AI, and 172 articles from Web of Science. In addition, 7 articles were obtained from backward screening of references from included studies. A total of 89 duplicates were identified and further excluded from this review. Screening of the title and abstract was conducted on 500 articles, and 214 articles were excluded. A total of 286 articles were eligible for full text evaluation. After full text evaluation, 114 articles were excluded because they did not fulfill the criteria of this study, i.e., the studies were not conducted on multidrug-resistant bacteria and the studies focused only on the mechanism of phage resistance (Figure 1). In total, 172 articles were included in this scoping review [14,15,16,17,18,19,20,21,22,23,24,25,26,27,28,29,30,31,32,33,34,35,36,37,38,39,40,41,42,43,44,45,46,47,48,49,50,51,52,53,54,55,56,57,58,59,60,61,62,63,64,65,66,67,68,69,70,71,72,73,74,75,76,77,78,79,80,81,82,83,84,85,86,87,88,89,90,91,92,93,94,95,96,97,98,99,100,101,102,103,104,105,106,107,108,109,110,111,112,113,114,115,116,117,118,119,120,121,122,123,124,125,126,127,128,129,130,131,132,133,134,135,136,137,138,139,140,141,142,143,144,145,146,147,148,149,150,151,152,153,154,155,156,157,158,159,160,161,162,163,164,165,166,167,168,169,170,171,172,173,174,175,176,177,178,179,180,181,182,183,184,185].

### 3.2. Types of Studies Included

**Figure 2:**
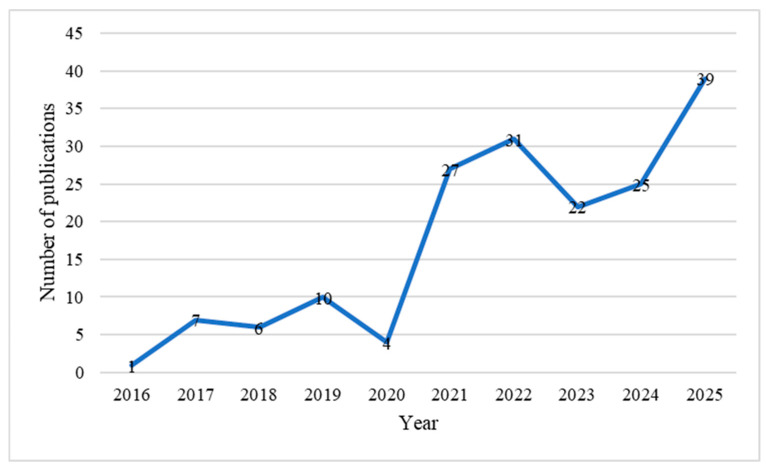
Trend on number of publications of phages as therapy from 2016 to 2025.

The majority of the 172 articles included in this scoping review were published in 2025 (n = 39 articles) while the lowest publication was observed in 2016 with only one article (Figure 2).

**Figure 3:**
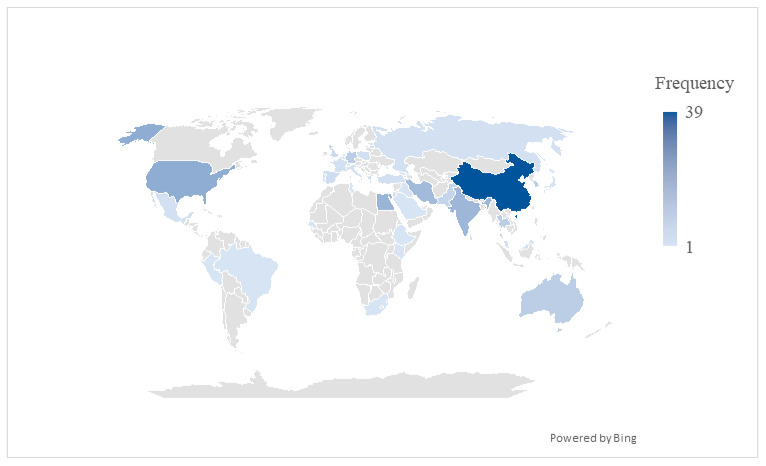
Number of publications of phage therapy based on countries.

From 172 articles, 65 studies were conducted on *K. pneumoniae*, followed by 55 studies on *P. aeruginosa*, and 48 studies on *A. baumannii*. Database search managed to capture four studies on *H. pylori*. However, no *H. pylori* study was found to be eligible for inclusion into this review as studies published did not explicitly mention antibiotic susceptibility of *H. pylori* isolates or study focused on lysogenic phage [186,187,188,189]. Meanwhile, two studies included both *K. pneumoniae* and *P. aeruginosa* in their studies, one study included both *P. aeruginosa* and *A. baumannii*, and one study included *K. pneumoniae*, *P. aeruginosa*, and *A. baumannii* in the published work. The articles were published from 37 countries. Most publications were from China (n = 39), followed by USA (n = 16), Egypt (n = 14), India (n = 13), and Iran (n = 12) (Figure 3).

### 3.3. Samples and Study Approach

Analysis of bacteriophage samples used in the studies reveals that phages were isolated from many sources, including sewage and wastewater, water sources that include rivers, ponds, and lakes, the library of phage collection, patient sputum, and soil. Most studies isolated phages from sewage and wastewater (n = 127), followed by environmental sources such as river, lake, ponds, and soil (n = l5), and library collection of phages readily available at either institute or pharmaceutical companies (n = 8). One study isolated a phage from a patient’s sputum. Sewage and wastewater from where phage samples obtained were located in hospital and urban areas. Nevertheless, 21 studies did not mention the sources of the phages used in their studies. Almost all studies (n = 167) named the phages isolated in their studies while five studies did not mention the names of phages isolated in their studies. For bacterial samples, 157 studies used multidrug-resistant bacteria, eight studies used extensively resistant bacteria, and six studies used pandrug-resistant bacteria. Of the eight studies that used extensively resistant bacteria, three studies each were conducted on *A. baumannii* and *P. aeruginosa*, and two studies were conducted on *K. pneumoniae*. Phage cocktail antibacterial activity was examined in 28 studies, while the rest of the studies (n = 138) only examined single phage antibacterial activity. Most studies (n = 126) used transmission electron microscopy and whole genome sequencing to identify morphology and type of phages isolated while 46 studies did not explicitly state the methods used to identify phages isolated in their studies.

**Figure 4:**
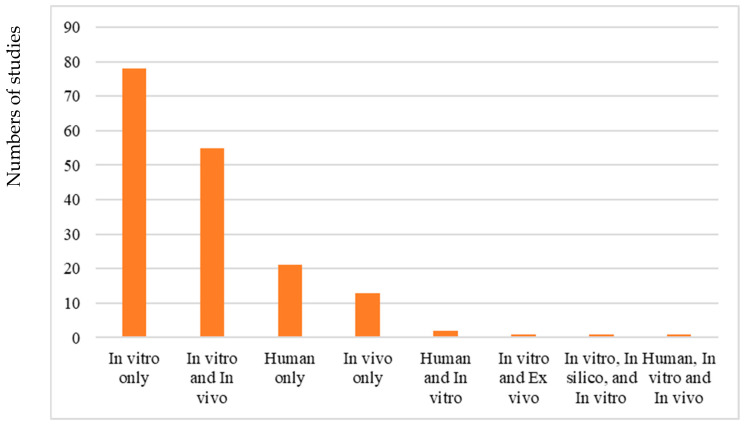
Study models used in bacteriophage studies.

Most studies used in vitro models only (n = 78), followed by in vitro and in vivo (n = 55), and human only (n = 21). Meanwhile, 13 studies used in vivo models only and two studies used human and in vitro models. One study each used in vitro and ex vivo model; and in vitro, in silico and in vitro model. Finally, one study used human, in vitro, and in vivo models in its study to examine phage efficacy. Studies that adopted an in vivo model used *Galleria mellonella*, zebrafish, rats, and mice in their studies. Of the human studies, four were conducted on *A. baumannii*, four on *K. pneumoniae*, and 13 on *P. aeruginosa*. One study examined both *A. baumannii* and *P. aeruginosa*. One study examined the efficacy of phages in patients infected with extensively drug-resistant *K. pneumoniae*, while three studies examined the efficacy of phage in patients infected with pandrug-resistant *K. pneumoniae* and *P. aeruginosa*, respectively. Interestingly, most studies that involved humans were reported as case studies and not large-scale trial (Figure 4).

### 3.4. Antibacterial Efficacy of Bacteriophages

Findings of all studies included in this scoping review are available in Supplementary Table S1. All studies included in this scoping review reveals that bacteriophages isolated in their studies had lytic activity against the bacteria tested. This finding indicates that the phages isolated have the potential to be used as a new therapeutic option for *K. pneumoniae*, *P. aeruginosa*, and *A. baumannii*. Furthermore, 29 studies used phage cocktails in their studies. Of 29 studies, four studies assessed the efficacy of the phage cocktail compared to single phage therapy and found the cocktail had better antibacterial activity than single phage [79,86,132,178]. In 46 studies, assessment of antibiofilm activity of phage against bacteria shows that the phages examined had antibiofilm activity against the bacteria examined. Of those studies, 17 studies were conducted on *P. aeruginosa*, 16 studies were conducted on *K. pneumoniae*, 12 studies were conducted on *A. baumannii* and one study was conducted on *K. pneumoniae* and *P. aeruginosa*.

Of note, 70 in vivo studies examined the efficacy of phage as alternative treatment for bacterial infection in animals. Out of 70 studies, 64 studies showed that phage administration managed to reduce bacterial load in animal models used while the rest of studies (n = 6) did not assess bacterial reduction parameter. Meanwhile, 32 out of 70 studies demonstrated that phage treatment cleared bacterial infection in animal models, while 37 studies did not explicitly mention bacterial clearance in animal models. One study observed that phage administration did not clear bacterial infection [157]. A total of 15 studies observed a reduction in inflammation in animal models. Of studies that observed bacterial clearance in animals, 31 studies (44.3%) infected the animals with *K. pneumoniae*, 20 (28.6%) infected the animals with *A. baumannii*, and the rest (n = 19, 27.1%) infected the animals with *P. aeruginosa*. In total, 8 studies that demonstrated bacterial clearance in animal models used a phage cocktail, and 7 studies also demonstrated that phages with bacterial clearance activity in animal models also had antibiofilm activity in vitro.

### 3.5. Antibacterial Efficacy of Bacteriophages in Human Study

Out of 172 studies included in this review, only 24 studies (14%) were conducted on humans. The majority of the studies (n = 21) were conducted on human subjects only without determination of phage antibacterial activity in vitro and in vivo models. Most studies (n = 21; 87.5%) reveal that phage administration to patients resulted in patients’ improvement and managed to clear bacterial infection at the infection sites. However, three studies reported that phage administration did not result in patient improvement. In the studies with negative report on phage administration to the patients, one study was conducted on patients infected with multidrug-resistant *A. baumannii*, one study was conducted on pandrug *P. aeruginosa*, while another study was conducted on patients infected with extended beta lactamase *K. pneumoniae*. Of the 21 studies that reported success in treatment using phage, 14 studies were conducted on *P. aeruginosa*, five studies were conducted on *K. pneumoniae*, four studies were conducted on *A. baumannii*, and one study was conducted on patients infected with MDR *P. aeruginosa* and *A. baumannii*. Notably, all studies that examined phage activity in humans had a small sample size and most were case study reports.

Table 1 summarizes the findings, including the dosage and route of administration in human studies. The 24 studies exhibit significant methodological heterogeneity in administration routes, primarily determined by the specific anatomical site of infection and the need to bypass biological barriers. While systemic intravenous (IV) therapy was used for bloodstream infections or sepsis, a majority of the research (37.5%) favored localized delivery methods, such as nebulization for respiratory issues, bladder irrigation, or direct instillation into surgical sites. Furthermore, 33% of the studies adopted a “multi-compartment” or dual-route approach—most commonly combining IV infusions with nebulization or percutaneous drains—to ensure high phage titers reached localized biofilms while simultaneously maintaining systemic coverage. Unusual delivery methods, such as oral “swish and swallow” techniques or combined oral and intra-rectal administration, further highlight the lack of a standardized delivery protocol in current clinical practice. The heterogeneity is equally pronounced in both dosing and potency, with concentrations spanning a range of approximately nine orders of magnitude (10^2^ to 10^11^ PFU/mL). Although most personalized salvage therapies targeted high concentrations between 10^8^ and 10^10^ PFU/mL to maximize efficacy, systemic IV doses were often constrained by safety rather than therapeutic goals. For example, some high-titer stocks were diluted to levels as low as 2.14 × 10^7^ PFU/mL to comply with strict FDA endotoxin thresholds. Conversely, one study demonstrated how manufacturing inconsistencies can lead to sub-therapeutic dosing, where a drop in titer to 10^2^ PFU/mL significantly hindered clinical outcomes. Temporal heterogeneity further complicates the landscape, as treatment durations ranged from a single intraoperative dose to prolonged courses lasting 86 days or even six months. Dosing frequency was similarly varied, with intervals ranging from once-daily applications to aggressive administration every two hours. Collectively, these findings suggest that while phage therapy offers high clinical flexibility, the extreme diversity in dosage and administration reflects a “personalized” phase of medicine that currently lacks universal guidelines, posing a challenge for standardized meta-analysis.

### 3.6. Mechanistic Insights

A total of six studies examined mechanistic insights of phage therapy [18,53,54,164,177,183]. Bacteriophage antibacterial activity is primarily mediated through specific receptor recognition, particularly targeting bacterial capsular polysaccharides (CPS). Phages utilize capsule components as primary binding sites, often aided by capsular depolymerases that degrade the capsule and facilitate access to the bacterial cell surface. However, this receptor specificity also drives the rapid emergence of resistance, commonly through mutations or disruptions in genes involved in capsule biosynthesis, namely *wbaP*, *wcaJ*, and *mshA*. These alterations result in capsule loss or structural modification, thereby preventing phage adsorption. Beyond capsule-associated changes, resistance mechanisms frequently involve broader remodeling of the bacterial cell envelope, including modifications to outer membrane proteins and lipooligosaccharide structures. Notably, these adaptations are often associated with significant biological trade-offs, including reduced bacterial fitness and virulence. Importantly, phage-resistant mutants have been shown to exhibit increased susceptibility to antibiotics, such as colistin, despite retaining their original resistance determinants, highlighting a phenomenon of collateral sensitivity. To address the rapid development of resistance, phage cocktail strategies have been proposed, as combining multiple phages targeting distinct receptors can delay resistance emergence and enhance antibacterial efficacy. Furthermore, evidence of horizontal transfer of receptor-binding domains among phages underscores their genetic adaptability and potential to expand host range. Collectively, these findings emphasize the dynamic interplay between phage infection, bacterial resistance, and evolutionary trade-offs, which can be strategically exploited to improve phage-based therapeutic approaches.

## 4. Discussion

The aims of this scoping review were to summarize the findings of the antibacterial efficacy of bacteriophages against multidrug-resistant *K. pneumoniae*, *P. aeruginosa*, *A. baumannii*, and *H. pylori*. We focus on lytic phage therapy because they directly kill the bacteria and low risk of virulence or resistance gene horizontal transfer [190]. Furthermore, this review aimed to identify gaps in studies published over a 10-year period. While most studies were on MDR *K. pneumoniae*, *P. aeruginosa*, and *A. baumannii*, *H. pylori* associated study were notably absent suggesting a paucity of bacteriophage research and discovery in this area of *H. pylori* research. This highlights a potential translational gap in the development of phage therapy for gastrointestinal infections, despite the global burden of *H. pylori*-associated disease [191]. One explanation for this paucity is because *H. pylori* is a fastidious and slow-growing organism which makes it difficult as a model organism in day-to-day research compared to other organisms which can be easily grown under aerobic conditions [192]. Although we managed to capture the literature on *H. pylori* lytic phage studies, the studies conducted were excluded from this review because they did not examine the antibiotic susceptibility of *H. pylori* isolates. This suggests there is a need for future studies on phage to examine resistance profiles of *H. pylori* for translation of findings to MDR *H. pylori*. We also observed an increasing year-to-year trend of the publications from 2015 to 2025, suggesting an increasing interest in adopting bacteriophage as a therapeutic option to combat the emergence of antibiotic resistance. In addition to that, we also found a lot of phage studies were from China, India, USA, Egypt, and Iran, which may reflect differences in research capacity, funding priorities, and the local burden of multidrug-resistant infections. Sewage and wastewater were highlighted as the main sources for lytic phages, underscoring their role as rich environmental reservoirs. However, inconsistent reporting of isolation sources across studies may affect reproducibility and comparability of findings.

Variable study models used in the publications were discovered. Most studies adopted single study model to examine antibacterial activity as demonstrated by in vitro studies. While one study model can be used to screen bacteriophage activity, a gap persists as phage efficacy in a culture model may not be translated to in vivo or human study. In addition, we identified there is no consensus in animal models used in phage studies. Type of animals used are different among studies. This heterogeneity may contribute to variability in reported outcomes due to differences in physiological, immunological, and microbiome-related complexity between models. This can result in variability in the data reported, as the animal models used differ in biological complexity [193]. Thus, there should be consensus on which model to be used for consistency. We also found that there is a paucity in adopting phage therapy in large scale human trials although studies included in this review reported positive result in human studies with small sample sizes. Patient selection, ethical regulation, clinical trial design, and phage delivery are usually factors that contribute to lack of large-scale human studies [194]. While most studies reported techniques used to determine the morphology and classification of phages isolated, some studies did not conduct the identification of the phage. Classification of phages is essential to determine the evolutionary and stability of phages for manufacturing, and to identify the presence of antibiotic resistance genes and virulence factors that may be transferred to the targeted bacteria [195].

All studies included in this review demonstrated lytic activity against the bacteria tested, suggesting their potential as a new therapeutic option to combat antibiotic resistance. Although studies examined the lytic activity of phage isolates, most did not examine the mechanisms of antibacterial activity, including antibiofilm activity, the specificity of phage, whether it is broad- or narrow-spectrum, and targeted receptors. Therefore, future research should also include an assessment of the antibacterial mechanism of phages. In studies that examined antibiofilm activity of phages, all of them showed that phage had antibiofilm activity against bacteria tested. Biofilm formation is essential for bacteria to evade antibiotic action and to persist on fomites. *P. aeruginosa* and *A. baumannii* use biofilm formation to aid their transmission in clinical settings [196]. Antibiofilm activity of phage against these clinically important bacteria can help to mitigate their transmission to immunocompromised patients in healthcare settings. Phage cocktail demonstrated better antibacterial activity compared to single phage in some studies. This is because a phage cocktail can target multiple bacterial strains, providing a broader spectrum of antibacterial activity than a single phage. Nevertheless, phage cocktail is more difficult to design as they have different stability and difficulty in phage optimization than that of single phage [197].

In animal models, almost all studies reveal that phage had antibacterial efficacy when administered to the animals. However, this result should be interpreted with caution as the studies published on phage efficacy in animal models used different types of animals with no consensus on which animal should be used for a phage study. Given different biological complexity in distinct types of animal models, there should be a consensus on which animal is the right model for a phage study [198]. In addition, a toxicity study on phage demonstrated to possess antibacterial activity against the bacteria tested should also be evaluated [198]. Even though phage is usually regarded as safe, a toxicity study of phage in animals ensures that phage formulation does not have a toxic effect on organs and tissues. It also evaluates the host’s immune response to phage prior to the human study. Of note, a lack of mechanistic studies in phage studies were also noticed. Although all studies isolated and tested phage antibacterial efficacy in vitro or in vivo, most studies did not examine the mechanisms of antibacterial efficacy. Bacterial phage resistance can also develop and should be addressed by studies that conduct antibacterial efficacy of phage.

For human studies, most studies demonstrated that administration phage to infected patients improved patient’s conditions. Nevertheless, a gap in data collection from human studies with large sample size remains a barrier in implementation of phage therapy in humans. The majority of the studies used small sample size of patients. Thus, a lack of data from large-scale clinical trials to evaluate phage efficacy poses a challenge to developing personalized phage therapy in patients and standardized regulatory requirements in humans. The studies also exhibit significant methodological heterogeneity, primarily driven by the anatomical site of infection. Administration routes vary from systemic intravenous therapy to localized delivery, such as nebulization or surgical instillation, with 33% of studies employing dual-route strategies. Dosage is equally inconsistent, spanning seven orders of magnitude. These titers are often constrained by endotoxin safety limits rather than biological requirements. Treatment durations varied widely, ranging from single intraoperative administrations to prolonged regimens lasting up to 86 days. This variability reflects a largely personalized ‘salvage’ approach, highlighting the current lack of standardized protocols necessary for universal clinical guidelines. Toxicity assessment should also be conducted to ensure safety of phage treatment in humans. In addition to that, pharmacogenomics can also be evaluated in human study for development of personalized medicine to the patients [199] and the application of artificial intelligence for screening potential lytic phages [200].

The advantages of this scoping review include comprehensive summary of updated knowledge on lytic phage therapy studies in MDR clinically important bacteria. We identified gaps in the studies across in vitro, animal models, and human studies. However, this scoping review also has limitations where we exclude bioengineered phage and lysogenic phage that may also have antibacterial efficacy against MDR pathogens. Furthermore, we excluded studies more than 10 years ago and studies not published in English that may demonstrate antibacterial efficacy against the pathogens.

## 5. Conclusions

This scoping review confirms the growing interest in lytic bacteriophages as a viable alternative against MDR *K. pneumoniae*, *P. aeruginosa*, and *A. baumannii*. Phages demonstrated clear lytic activity across all studies, with phage cocktails and antibiofilm activity showing superior results in in vitro and in vivo animal models. While most human case studies reported patient improvement, significant gaps remain, notably the absence of research on MDR *H. pylori* and the urgency to implement studies using a large sample size. Future research should prioritize larger, well-designed clinical trials and standardized experimental models. Emerging tools such as artificial intelligence may accelerate phage discovery, host–phage matching, and phage cocktail design. In parallel, stronger regulatory frameworks, national phage banks, and international collaboration will be essential to support the translation of phage therapy into routine clinical practice in the fight against antimicrobial resistance. Future research must focus on standardizing animal models, fully characterizing antibacterial mechanisms, and, most critically, transitioning from small case reports to large-scale, well-designed human clinical trials to achieve widespread clinical implementation.

## Figures and Tables

**Table 1:** Summary of phage therapy heterogeneity (n = 24) in human studies.

Ref.	Author (Year)	Route of Administration	Dosage/Concentration	Duration
[26]	Aslam (2019)	Dual: IV + Nebulized	10^7^ to 5 × 10^9^ PFU/mL	Variable
[27]	Aslam (2020)	Dual: IV + Nebulization/Instillation	Variable doses	Several weeks
[30]	Bao (2020)	Local: Bladder irrigation	5 × 10^8^ PFU/mL (50 mL)	5 days
[35]	Chan (2025)	Local: Inhaled (Nebulized)	1 × 10^10^ PFU per session	7–10 days
[40]	Chen (2022)	Dual: Nebulized + Intrapleural	Not specified	24 days
[45]	Corbellino (2019)	Dual: Oral + Intra-rectal	Personalized (not detailed)	Initiated December 2017
[51]	Eskenazi (2022)	Local: Percutaneous catheter	Max concentration	6 days
[61]	Ferry (2022)	Dual: IV + Tissue Instillation	IV: 10^6^ PFU/mL	21 days
[70]	Hahn (2023)	Local: Inhaled (Nebulized)	1 × 10^10^ PFU/mL	7–9 days
[74]	Jault (2019)	Local: Topical (Wound dressing)	Low: 1 × 10^2^ PFU/mL	7 days
[78]	Jernigan (2025)	Local: Oral (“Swish and swallow”)	1–2 ampules	6 months
[84]	Kohler (2023)	Local: Inhaled (Aerosolized)	5 × 10^9^ PFU per dose	7 days (total)
[87]	Kuipers (2019)	Dual: Oral + Intravesical	Standard vials (unreported PFU)	8 weeks
[89]	LaVergne (2018)	Systemic: IV	Diluted: 2.14 × 10^7^ PFU/mL	8 days
[113]	Munteanu (2025)	Local: Joint washout/Drains	10^8^ PFU/mL	Single dose to 4 days
[130]	Racenis (2023)	Dual: IV + Topical	10^6^ to 10^11^ PFU/mL (range)	Concurrent with surgery
[131]	Racenis (2022)	Local: Bone/Wound bed instillation	2.5 × 10^9^ PFU/mL	Intraoperative
[133]	Rao (2022)	Dual: IV + Nebulized	1 × 10^9^ PFU/mL	7 days
[139]	Schooley (2017)	Dual: IV + Percutaneous	Diluted based on endotoxin limits	8 weeks
[155]	Tan (2021)	Local: Inhaled (Nebulized)	Personalized high concentration	16 days
[156]	Teney (2024)	Dual: IV + Nebulized	Not detailed	Daily (duration not specified)
[159]	Tkhilaishvili (2019)	Local: Intra-joint via drains	10^8^ PFU/mL	5 days
[163]	Van Nieuwenhuyse (2022)	Systemic: IV	Personalized	86 days
[175]	Yang (2025)	Local: Inhaled (Nebulization)	High-titer active levels	8 weeks

## Data Availability

No new data were created or analyzed in this study. Data sharing is not applicable to this article.

## Supplementary Materials

The following supporting information can be downloaded at: https://www.mdpi.com/article/10.3390/ph19050727/s1, Table S1: Summary of articles included in this study.
