# Supplementary material for: Phage Therapy in Combating Multidrug-Resistant Gram-Negative Pathogens: A Scoping Review
Source: Pharmaceuticals (Basel). 2026 May 3;19(5):727. doi: 10.3390/ph19050727 (PMC13209962; doi:10.3390/ph19050727)
Supplement: Supplementary file 1 [file pharmaceuticals-19-00727-s001.zip › pharmaceuticals-4235600-supplementary.pdf]

Supplementary Table 1 (Table S1): Summary of articles included in the study

Ref No.	Name of author	Year	Title	Country	Phage name	Sample source	Bacteria tested	Finding (concise)
14	Abdel-Razek et al.	2025	Analysis of a novel phage as a promising biological agent targeting multidrug resistant Klebsiella pneumoniae	Egypt	vB_KpnP_KP17	Sewage	KP	Sewage is reliable for isolating Pseudomonas phages; hospital sewage yields efficient phages, while river phages target resistant strains.
15	Abebe et al.	2025	Isolation, purification, and phenotypic characterization of virulent Klebsiella pneumoniae phages from environmental samples in Addis Ababa, Ethiopia: A synergistic approach combining spot assay and streak plating	Ethiopia	22 phages, but 8 phages were selected further for testing that include GGMp-TASP18, AKSp-TASP18, KIDp-TASP92, TTMpTASP92, GKMs-TASP18, AKSs-TASP18, TTMs -TASP92, and ADMs-TASP92	sewage and wastewater samples	KP	A combined purification method efficiently isolated 22 phages, yielding eight highly stable and active phages with broad host ranges.
16	Aghaee et al.	2021	Improving the Inhibitory Effect of Phages against Pseudomonas aeruginosa Isolated from a Burn Patient Using a Combination of Phages and Antibiotics	Iran	16 lytic phages, but some phages namely vB_PaeM_GUMS6, vB_PaeM_GUMS32, and vB_PaeM_GUMS45 were used	(three phages), hospital sewage (four phages), and a local river	PA	A combination of two phages and one antibiotic showed the highest killing efficiency against multidrug-resistant P.

						(nine phages).		aeruginosa, supporting the potential of phage-antibiotic cocktails at sub-MIC levels.
17	Aghaee et al.	2021	Sewage and sewage-contaminated environments are the most prominent sources to isolate phages against Pseudomonas aeruginosa	Iran	18 isolated phages (including three were named: vB_PaeM_GUMS6, vB_PaeM_GUMS32, and vB_PaeM_GUMS45)	hospital sewage, river, and soil	PA	Sewage and hospital environments are the most reliable sources for isolating highly efficient Pseudomonas phages, while river phages target strains with higher antibiotic resistance.
18	Ajakkala et al.	2024	Phenotypic Changes in Phage Survivors of Multidrug-Resistant Klebsiella pneumoniae	India	ØKp11 and ØKp26	water and soil samples	KP	Survivors of phages ØKp11 and ØKp26 showed reduced growth, impaired biofilm formation, altered antibiotic sensitivity, and decreased OMPs

								expression compared to the parent MDRKP002 isolate.
19	Akremi et al.	2022	Isolation and Characterization of Lytic Pseudomonas aeruginosa Bacteriophages Isolated from Sewage Samples from Tunisia	Tunisia	PsCh, PsIn, Ps25, and Ps12on-D	sewage	PA	Phage Ps12on-D exhibited a short latency period, large burst size, and broad stability across temperatures and pH levels, effectively lysing various P. aeruginosa strains worldwide.
20	Al-Madboly et al.	2023	Characterization and genomic analysis of novel bacteriophage NK20 to revert colistin resistance and combat pandrug-resistant Klebsiella pneumoniae in a rat respiratory infection model	Egypt	NK20	Sewage	KP	Intranasal administration of phage NK20 significantly reduced bacterial load and inflammation, rescuing 100% of rats challenged with pandrug-resistant K. pneumoniae.
21	Alseth et al.	2025	Mystique, a broad host range Acinetobacter phage, reveals the impact of culturing	USA	Mystique	Sewage	AB	Phage Mystique demonstrated a broad host range

			conditions on phage isolation and infectivity					against A. baumannii, with infectivity increasing from 85.4% on solid media to 91.3% in liquid culture.
22	Gordillo Altamirano et al.	2022	Phage-antibiotic combination is a superior treatment against Acinetobacter baumannii in a preclinical study	Australia	øFG02 and øLK01	Sewage	AB	Phage øFG02 drives the in vivo evolution of A. baumannii towards a capsule-deficient, phage-resistant phenotype that is resensitized to ceftazidime.
23	Arumugam et al.	2022	Antibacterial efficacy of lytic phages against multidrug-resistant Pseudomonas aeruginosa infections in bacteraemia mice models	India	AP025 and Pseudomonas phage AP006	Sewage samples from Bangalore	PA	A single dose of phages at higher concentrations effectively eliminated bloodstream infections and achieved 100% survival in mice.
24	Asif et al.	2023	A K-17 serotype specific Klebsiella phage JKP2 with biofilm reduction potential	Pakistan	JKP2	Sewage	KP	Phage JKP2 specifically targets the K. pneumoniae K-17 serotype, demonstrating stability and

								efficient elimination of both planktonic cells and mature biofilms.
25	Askoura et al.	2021	Characterization of Polyvalent Bacteriophages Targeting Multidrug-Resistant Klebsiella pneumonia with Enhanced Anti-Biofilm Activity	Egypt	ΦKpnM-vB1 and ΦKpnM-vB3, ΦKpnP-vB2	Sewage samples	KP	Isolated phages ΦKpnM-vB1, ΦKpnP-vB2, and ΦKpnM-vB3 showed high stability and lytic activity, efficiently reducing Klebsiella biofilm formation.
26	Aslam et al.	2019	Early clinical experience of bacteriophage therapy in 3 lung transplant recipients	USA	AmpliPhi Biosciences Corporation (San Diego, CA), Naval Medical Research Center (Fort Detrick, Maryland) and Adaptive Phage Therapeutics (Gaithersburg, MD).	Not mentioned	PA	Bacteriophage therapy was well tolerated and associated with clinical improvement in lung transplant recipients with MDR bacterial infections unresponsive to antibiotics alone.
27	Aslam et al.	2020	Lessons Learned From the First 10 Consecutive Cases of Intravenous Bacteriophage Therapy to Treat Multidrug-Resistant Bacterial Infections	USA	Wide range of bacteriophage types	Not mentioned	PA, AB	Successful treatment was achieved in four patients infected

			at a Single Center in the United States					with P. aeruginosa and A. baumannii.
28	Assafiri et al.	2021	Klebsiella virus UPM2146 lyses multiple drug-resistant Klebsiella pneumoniae in vitro and in vivo	Malaysia	UPM2146	Polluted lake	KP	Phage UPM2146 demonstrated high lytic activity against K. pneumoniae and proved safe and effective for treatment in a zebrafish larvae model.
29	Baginska et al.	2023	Biological Properties of 12 Newly Isolated Acinetobacter baumannii-Specific Bacteriophages	Poland	12 different types of phages were isolated	environmental, municipal, and hospital wastewater samples	AB	Twelve A. baumannii-specific phages were isolated, mostly temperate siphoviruses, exhibiting broad lytic spectra and varying optimal infection parameters.
30	Bao et al.	2020	Non-active antibiotic and bacteriophage synergism to successfully treat recurrent urinary tract infection caused by extensively drug-resistant Klebsiella pneumoniae	China	Cocktail 1:Five lytic phages, namely, SZ-1, SZ-2, SZ-3, SZ-6, and SZ-8 and cocktail 2:Kp165, Kp166, Kp167, Kp158, and Kp169	Not mentioned	KP	The combination of sulfamethoxazole-trimethoprim with a phage cocktail successfully cured a patient's UTI and inhibited the emergence of

								phage-resistant mutants in vitro.
31	Baqer et al.	202 2	In Vitro Activity, Stability and Molecular Characterization of Eight Potent Bacteriophages Infecting Carbapenem-Resistant Klebsiella pneumoniae	Malaysia	Eight bacteriophages	wastewater, soil and clams	KP	All tested bacteriophages rapidly reduced the optical density of planktonic K. pneumoniae, indicating significant potential to control infections.
32	Camens et al.	202 1	Preclinical Development of a Bacteriophage Cocktail for Treating Multidrug Resistant Pseudomonas aeruginosa Infections	Australia	PA4	Wastewater samples	PA	Phage PA4 exhibited broad infectivity, stability, and lytic ability against clinical isolates, suggesting it is a strong candidate for treating P. aeruginosa infections.
33	Cao et al.	202 5	Evaluation of the efficacy of a bacteriophage in the treatment of pneumonia induced by multidrug resistance Klebsiella pneumoniae in mice	China	phage 1513	sewage samples	KP	Phage 1513 demonstrated significant in vitro and in vivo efficacy, protecting mice from lethal pneumonia caused by multidrug-

								resistant K. pneumoniae.
34	Cha et al.	2018	Characterization of Two Novel Bacteriophages Infecting Multidrug-Resistant (MDR) Acinetobacter baumannii and Evaluation of Their Therapeutic Efficacy in Vivo	SK	PBAB08 and PBAB25	Sewage sample	AB	A cocktail of two newly isolated phages significantly improved the survival rate and reduced bacterial load in mice infected with A. baumannii.
35	Chan et al.	2025	Personalized inhaled bacteriophage therapy for treatment of multidrug-resistant Pseudomonas aeruginosa in cystic fibrosis	USA	Not mentioned	Not mentioned	PA	Personalized nebulized phage therapy reduced sputum Pseudomonas density and improved lung function in cystic fibrosis patients without altering the microbiome.
36	Chang et al.	2022	The effects of different doses of inhaled bacteriophage therapy for Pseudomonas aeruginosa pulmonary infections in mice	Australia	PEV31	Sewage	PA	Pulmonary delivery of phage PEV31 significantly reduced bacterial load and suppressed inflammatory cytokines in mice,

								though higher doses led to increased phage-resistant mutants.
37	Chaplin et al.	2025	Pseudomonas Phage Banzai: Genomic and Functional Analysis of Novel Pbunavirus with Lytic Activity Against Pseudomonas aeruginosa	Russia	phage Banzai	Water sample from pond	PA	Phage Banzai, a novel Pbunavirus, demonstrated genomic stability, strict lytic activity, and significant therapeutic efficacy against P. aeruginosa in a Galleria mellonella model.
38	Chen et al.	2025	Genomic analysis and therapeutic efficacy evaluation of bacteriophage PK2420 for pneumonia caused by hypervirulent Klebsiella pneumoniae (K20 serotype)	China	Phage PK2420	hospital sewage	KP	Phage PK2420 rapidly lyses hypervirulent K. pneumoniae, inhibits biofilm formation, and significantly improves survival rates in murine pneumonia models without observable side effects.
39	Chen et al.	2017	Clinical Antibiotic-resistant Acinetobacter baumannii Strains with Higher Susceptibility to	Taiwan	24 active phages	sewage, park ponds, lotus farms,	AB	Antibiotic-resistant A. baumannii strains exhibited

			Environmental Phages than Antibiotic-sensitive Strains			or river water		significantly higher susceptibility to environmental phages compared to antibiotic-sensitive strains, indicating an evolutionary trade-off.
40	Chen et al.	2022	Bacteriophage therapy for empyema caused by carbapenem-resistant Pseudomonas aeruginosa	China	PA3 and PA18	Not mentioned	PA	A personalized two-phage preparation combined with conventional antibiotics effectively alleviated a multidrug-resistant P. aeruginosa infection in a patient with empyema.
41	Choi et al.	2024	Synergistic Antimicrobial Effects of Phage vB_AbaSi_W9 and Antibiotics against Acinetobacter baumannii Infection	SK	vB_AbaSi_W9	Sewage	AB	Phage vB_AbaSi_W9 demonstrated excellent synergistic effects against carbapenem-resistant A.

								baumannii when combined with tigecycline and rifampicin, achieving a 100% survival rate in a mouse model.
42	Choi et al.	202 5	Overcoming phage resistance: efficacy of sequential phage-colistin therapy against carbapenem- resistant Acinetobacter baumannii	SK	vB_AbaSt_W16	sewage	AB	Sequential administration of colistin after phage treatment significantly improved bacterial clearance, enhanced survival, and suppressed resistance emergence in CRAB infections.
43	Chow et al.	202 0	Pharmacokinetics and Time-Kill Study of Inhaled Antipseudomonal Bacteriophage Therapy in Mice	Australia	PEV31	sewage	PA	Pulmonary delivery of phage PEV31 in mice successfully suppressed bacterial growth and reduced the MDR bacterial burden, despite the emergence of some phage-resistant isolates.

44	Cieslik et al.	2025	Biological characterization and stability of three lytic wastewater-derived bacteriophages targeting multidrug-resistant Acinetobacter baumannii and A. johnsonii clinical strains	Poland	Acba_19, Acjo_20 and Acba_21	Sewage	AB	A cocktail of three newly isolated Acinetobacter phages lysed susceptible strains without demonstrating synergistic effects or phage interference.
45	Corbellino et al.	2019	Eradication of a Multidrug-Resistant, Carbapenemase-Producing Klebsiella pneumoniae Isolate Following Oral and Intra-rectal Therapy With a Custom Made, Lytic Bacteriophage Preparation	Italy	Not mentioned	Not mentioned	KP	A custom-made lytic bacteriophage preparation successfully eradicated a multidrug-resistant K. pneumoniae infection in a patient without adverse effects.
46	Duplessis	2021	Successful Intratracheal Treatment of Phage and Antibiotic Combination Therapy of a Multi-Drug Resistant Pseudomonas aeruginosa Murine Model	USA	PsA (PaAH2ΦP (103), PaBAP5Φ2 (130), and PaΦ (134))	Naval Medical Research Center Biological Defense Research Directorate (BDRD) current	PA	Intratracheal administration of phages protected mice from lethal infection, and partial additive effects were observed when combined with a sub-efficacious

						library of phages collected from various environment al sources		dose of meropenem.
47	Ebrahimi et al.	202 1	Efficacy of isolated bacteriophage against biofilm embedded colistin-resistant Acinetobacter baumannii	Iran	IsfAB78	Wastewater	AB	The isolated lytic phage IsfAB78 significantly reduced biofilm formation by up to 87% in MDR A. baumannii clinical isolates.
48	El-Din et al.	202 5	Bacteriophage-antibiotic synergy enhances therapeutic efficacy against multidrug-resistant Klebsiella pneumoniae infections	Egypt	vB_Kpn_FOPMU1	Not mentioned	KP	The combination of bacteriophage and cefotaxime demonstrated strong synergistic activity, enhancing bacterial clearance and achieving a 100% survival rate in a murine K. pneumoniae lung infection model.
49	Engeman et al.	202 1	Synergistic Killing and Re-Sensitization of Pseudomonas aeruginosa to Antibiotics by Phage-Antibiotic Combination Treatment	USA	Phage cocktail:EPa5, EPa11, EPa15, EPa22, and EPa43	Not mentioned	PA	Treatment with phage PAM2H combined with antibiotics re-sensitized P.

								aeruginosa to antibiotics in vitro and synergistically reduced bacterial burden in a mouse wound model.
50	Erol et al.	2021	The effect of phage-antibiotic combination strategy on multidrug-resistant Acinetobacter baumannii biofilms	Turkey	C2 phage, K3 phage and phage cocktail (C2 + K3 phage)	Not mentioned	AB	The combination of phages and antibiotics, particularly when applied sequentially, was more effective than single applications in eradicating mature biofilms of antibiotic-resistant bacteria.
51	Eskenazi et al.	2022	Combination of pre-adapted bacteriophage therapy and antibiotics for treatment of fracture-related infection due to pandrug-resistant Klebsiella pneumoniae	Belgium	vB_KpnM_M1	sewage	KP	A pre-adapted bacteriophage combined with antibiotics successfully treated a pandrug-resistant K. pneumoniae infection in a patient with a fracture-related wound.

52	Essam et al.	2025	Isolation and characterization of phages ΦZC2 and ΦZC3 against carbapenem-resistant Acinetobacter baumannii, and efficacy of ΦZC3 on A549 cells	Egypt	ΦZC2 and ΦZC3	hospital wastewater	AB	Phages ΦZC2 and ΦZC3 demonstrated stability and broad host ranges, with ΦZC3 successfully rescuing lung cells from MDR A. baumannii infection without cytotoxicity.
53	Fang et al.	2022	Lytic Phages against ST11 K47 Carbapenem-Resistant Klebsiella pneumoniae and the Corresponding Phage Resistance Mechanisms	China	P13	Hospital sewage	KP	Phage P13 is a novel, stable lytic phage against CRKP, though the rapid emergence of resistant mutants highlights the need for diverse phage cocktails.
54	Fang et al.	2022	Characterization of phage resistance and phages capable of intestinal decolonization of carbapenem-resistant Klebsiella pneumoniae in mice	China	P24 and P39	Sewage	KP	Lytic phages P24 and P39 significantly decreased bacterial load in mouse intestinal colonization models, demonstrating their potential for

								decolonizing CRKP.
55	Farooq et al.	202 5	A pharmacometric model assessing the in vitro synergistic effect of a bacteriophage-polymyxin B combination in a clinical multidrug-resistant Klebsiella pneumoniae isolate	Germany and Australia	pK8	Sewage	KP	The combined application of phage pK8 and polymyxin B resulted in notable bactericidal effects and complete eradication of K. pneumoniae by resensitizing the bacteria to the antibiotic.
56	Fayez et al.	202 1	Topically Applied Bacteriophage to Control Multi-Drug Resistant Klebsiella pneumoniae Infected Wound in a Rat Model	Egypt	ZCKP8	Sewage	KP	Phage ZCKP8 demonstrated high stability and dose-dependent inhibition of K. pneumoniae in vitro, and significantly improved wound healing in an infected rat model.

57	Fayez et al.	202 3	Morphological, biological, and genomic characterization of Klebsiella pneumoniae phage vB_Kpn_ZC2	Egypt	ZCKP2	sewage	KP	Phage ZCKP2 is a safe and efficient candidate for phage therapy against multidrug-resistant K. pneumoniae, demonstrating high stability and consistent lytic activity.
58	Feng et al.	202 3	Characterization and genome analysis of phage vB_KpnS_SXFY507 against Klebsiella pneumoniae and efficacy	China	vB_KpnS_SXFY507	sewage	KP	Phage vB_KpnS_SXFY5 07 exhibited broad pH and thermal stability,

			assessment in Galleria mellonella larvae					significant in vitro antibacterial activity, and increased the survival rate of infected G. mellonella larvae.
59	Feng et al.	202 4	Safety and efficacy of a phage cocktail on murine wound infections caused by carbapenem-resistant Klebsiella pneumoniae	China	P24, P39 and PH1	Not mentioned	KP	Phage treatment significantly decreased wound bacterial load and accelerated healing in infected mice without any observed adverse events.
60	Ferriol-González et al.	202 4	Targeted phage hunting to specific Klebsiella pneumoniae clinical isolates is an efficient antibiotic resistance and infection control strategy	Spain	83 phages isolation	wastewater treatment plants and surrounding areas in Valencia (Spain)	KP	A 12-phage cocktail was effective against 31% of carbapenem-resistant K. pneumoniae isolates, highlighting the need for specific phage-hunting strategies for highly variable encapsulated hosts.

61	Ferry et al.	2022	Personalized bacteriophage therapy to treat pandrug-resistant spinal Pseudomonas aeruginosa infection	France	B_PaeP_4029, vB_PaeP_4032, and vB_PaeP_4034	Not mentioned	PA	A patient with a pandrug-resistant P. aeruginosa spinal abscess was successfully treated using a combination of surgery and personalized phage therapy.

62	Fong et al.	2017	Activity of Bacteriophages in Removing Biofilms of Pseudomonas aeruginosa Isolates from Chronic Rhinosinusitis Patients	Australia	4 anti-P. aeruginosa bacteriophages (Pa 193, Pa 204, Pa 222, Pa 223), as well as heat-inactivated stocks, were supplied by AmpliPhi Biosciences	Company	PA	Phage treatment significantly reduced biofilm biomass across various P. aeruginosa strains, accompanied by a rise in phage titers.

63	Forti et al.	201 8	Design of a Broad-Range Bacteriophage Cocktail That Reduces Pseudomonas aeruginosa Biofilms and Treats Acute Infections in Two Animal Models		PYO2, DEV, E215, and E217,	Sewage and previously characterize d phages	PA	A rationally designed 6-phage cocktail effectively targeted MDR P. aeruginosa, disrupted biofilms, and successfully treated infections in animal models.
64	Gan et al.	202 2	Bacteriophage Effectively Rescues Pneumonia Caused by Prevalent Multidrug-Resistant Klebsiella pneumoniae in the Early Stage	China	pKp11 and pKp383	hospital sewage	KP	Phages pKp11 and pKp383 provided effective treatment for early-stage pneumonia in mice, with their

								cocktail showing enhanced reduction in bacterial loads and inflammation.
65	Ghajavand et al.	2017	Isolation of bacteriophages against multidrug resistant Acinetobacter baumannii	Iran	IsfAB78 and IsfAB39	hospital wastewater	AB	Isolated phages with narrow host ranges significantly decreased A. baumannii turbidity, indicating their potential as candidates for phage therapy.
66	Ghanaim et al.	2023	Bacteriophage therapy as an alternative technique for treatment of multidrug-resistant bacteria causing diabetic foot infection	Egypt	Ps1	sewage	PA	A phage cocktail demonstrated superior efficacy over ceftriaxone in improving wound healing parameters and reducing inflammation in diabetic infected wounds.
67	Ghaznavi-Rad et al.	2022	Isolation of a lytic bacteriophage against extensively drug-resistant Acinetobacter baumannii infections and its dramatic	Iran	vB-AbauM-Arak1	wastewater	AB	Phage vB-AbauM-Arak1 exhibited high specificity and stability, significantly decreasing the

			effect in rat model of burn infection					lesion area in phage-treated groups compared to untreated controls.
68	Gou et al.	2025	Potential of a phage cocktail in the treatment of multidrug-resistant Klebsiella pneumoniae pulmonary infection in mice	China	GZ7 and GZ9	hospital sewage	KP	A phage cocktail of GZ7 and GZ9 effectively inhibited K. pneumoniae growth in vitro and significantly improved lung lesion conditions and survival in a mouse pulmonary infection model.
69	Guo et al.	2025	Identification and preclinical efficacy evaluation of two lytic bacteriophages targeting highly virulent and multidrug-resistant Klebsiella pneumoniae	China	hages vB_KpnP_XY3 and vB_KpnP_XY4	hospital environment	KP	Phage-antibiotic combinations significantly reduced bacterial loads and attenuated inflammation in murine pneumonia models, outperforming antibiotics alone.

70	Hahn et al.	2023	Bacteriophage Therapy for Pan-Drug-Resistant Pseudomonas aeruginosa in Two Persons With Cystic Fibrosis	USA	INF and pB	Phage library	PA	Personalized inhaled phage therapy proved safe and provided short-term clinical benefits, but failed to achieve sustained bacterial clearance in severe, late-stage CF patients.

71	Hesse et al.	2021	Bacteriophage Treatment Rescues Mice Infected with Multidrug-Resistant Klebsiella pneumoniae ST258	USA	P1 and P2	Sewage	KP	Systemic phage therapy effectively treated MDR K. pneumoniae bloodstream infections in vivo, improving survival and reducing bacterial burden depending on timing and host factors.

72	Hussain et al.	2021	Investigations on Acinetophage, QAB 3.4, Targeting Extensively Drug-Resistant Acinetobacter baumannii Isolates	Pakistan	QAB 3.4	Sewage	AB	Sewage-derived phage QAB 3.4 demonstrated broad activity and strong in vitro antibacterial effects against MDR A. baumannii, indicating potential as a surface disinfectant.
73	Jansen et al.	2018	Enhanced antibacterial effect of the novel T4-like bacteriophage KARL-1 in combination with antibiotics against multi-drug resistant Acinetobacter baumannii	Germany	vB_AbaM-KARL-1	Aquatic samples from university hospital	AB	Phage KARL-1 achieved complete clearance of liquid cultures when combined with meropenem, demonstrating significantly augmented antibacterial activity.
74	Jault et al.	2019	Efficacy and tolerability of a cocktail of bacteriophages to	Germany	Cocktail of lytic phages	Not mentioned	PA	Phage PP1131 decreased

			treat burn wounds infected by Pseudomonas aeruginosa (PhagoBurn): a randomised, controlled, double-blind phase 1/2 trial					bacterial burden in burn wounds at a slower pace than standard care, warranting further studies with higher concentrations.
75	Jeon et al.	201 9	Efficacy of bacteriophage treatment against carbapenem-resistant Acinetobacter baumannii in Galleria mellonella larvae and a mouse model of acute pneumonia	SK	Bϕ-R2096	sewage	AB	Phage Bϕ-R2096 exhibited strong bacteriolytic activity, significantly increased survival rates in in vivo models, and ameliorated histologic damage in infected lungs without side effects.
76	Jeon et al.	201 6	In Vivo Application of Bacteriophage as a Potential Therapeutic Agent To Control OXA-66-Like Carbapenemase-Producing Acinetobacter baumannii Strains Belonging to Sequence Type 357	SK	phage Bϕ-C62	Sewage	AB	Phage Bϕ-C62 displayed high stability and strong cell lysis activity, successfully clearing lung infections and ensuring 100% survival in a

								mouse intranasal infection model.
77	Jeon et al.	2019	Two Novel Bacteriophages Improve Survival in Galleria mellonella Infection and Mouse Acute Pneumonia Models Infected with Extensively Drug-Resistant Pseudomonas aeruginosa	SK	Bϕ-R656 and Bϕ-R1836	Hospital sewage	PA	Phages Bϕ-R656 and Bϕ-R1836 exhibited broad host ranges and strong bacteriolytic activity, significantly enhancing survival and decreasing bacterial load in in vivo infection models.
78	Jernigan et al.	2025	Successful Treatment of a Patient With Chronic Bronchiectasis Using an Induced Native Phage Cocktail: A Case Report	USA	native monovalent or polyvalent phages in a highly precise broad-spectrum cocktail	Not mentioned	PA	Induced native phage cocktails demonstrated rapid, gentle, and effective long-term results in treating a patient with severe non-cystic fibrosis bronchiectasis.
79	Jokar et al.	2023	Antibacterial effects of single phage and phage cocktail against multidrug-resistant Klebsiella pneumoniae isolated from diabetic foot ulcer	Iran	KP1, KP2, KP3, and KP4	Sewage	KP	A phage cocktail demonstrated significantly higher antibacterial activity than single phages,

								successfully eradicating MDR K. pneumoniae without allowing bacterial regrowth.
80	Karthika et al.	2023	Two novel phages PSPa and APPa inhibit planktonic, sessile and persister populations of Pseudomonas aeruginosa, and mitigate its virulence in Zebrafish model	India	PSPa and APPa	Sewage and pond	PA	Two identified phages exhibited high host specificity, showing antagonistic activity against the vast majority of MDR clinical isolates of P. aeruginosa.
81	Kelishomi et al.	2024	Evaluation of the therapeutic effect of a novel bacteriophage in the healing process of infected wounds with Klebsiella pneumoniae in mice	Iran	Not mentioned	Sewage	KP	An isolated Drexlerviridae phage effectively eliminated bacteria from wounds, resulting in better physical condition for mice in the phage therapy group.
82	Kifelew et al.	2024	Lytic activity of phages against bacterial pathogens infecting diabetic foot ulcers	Australia	AB-PA01 (4-phage cocktail)	AmpliPhi Australia Pty Ltd	PA	Phage treatments AB-SA01 and AB-PA01 significantly reduced biofilm biomass of their

								hosts in the planktonic state, regardless of antibiotic resistance characteristics.
83	Kim et al.	2024	A blueprint for broadly effective bacteriophage-antibiotic cocktails against bacterial infections	USA	Phage cocktail	Not mentioned	PA	Three phage-antibiotic cocktails demonstrated high efficacy against most P. aeruginosa clinical isolates and showed comparable effectiveness in an in vivo wound infection model.
84	Kohler et al.	2023	Personalized aerosolised bacteriophage treatment of a chronic lung infection due to multidrug-resistant Pseudomonas aeruginosa	Switzerland	vFB297	Not mentioned	PA	Personalized aerosolized phage therapy resulted in significant clinical improvement for a patient with a chronic MDR P. aeruginosa infection, despite the emergence of phenotypically diverse strains.
85	Kondo et al.	2023	Characterization of 29 newly isolated bacteriophages as a potential therapeutic agent	Japan	29 phages	sewage	KP	A 10-phage cocktail effectively

			against IMP-6-producing Klebsiella pneumoniae from clinical specimens					delayed the emergence of phage-resistant K. pneumoniae, demonstrating the potential of well-designed cocktails in mitigating resistance.
86	Kovacs et al.	2024	Combinations of Bacteriophage Are Efficacious against Multidrug-Resistant Pseudomonas aeruginosa and Enhance Sensitivity to Carbapenem Antibiotics	USA	Phages PaPC1, PaWP1, and PaWP2	sewage	PA	A cocktail of three lytic phages significantly disrupted biofilms and completely inhibited the growth of MDR P. aeruginosa, showing broad efficacy across diverse clinical strains.
87	Kuipers et al.	2019	A Dutch Case Report of Successful Treatment of Chronic Relapsing Urinary Tract Infection with Bacteriophages in a Renal Transplant Patient	Netherlands	Eliava Institute	Not mentioned	KP	A relapsing ESBL-positive Gram-negative infection in a renal transplant patient was successfully treated with a combination of meropenem and bacteriophages.

88	Latz et al.	2017	Differential Effect of Newly Isolated Phages Belonging to PB1-Like, phiKZ-Like and LUZ24-Like Viruses against Multi-Drug Resistant Pseudomonas aeruginosa under Varying Growth Conditions	Germany	SL1 SL2, and SL4	Hospital sewage	PA	Selected phages successfully suppressed planktonic cultures and rescued bacteria-infected wax moth larvae, though no synergistic effects were observed among the phages in a cocktail.
89	LaVergne et al.	2018	Phage Therapy for a Multidrug-Resistant Acinetobacter baumannii Craniectomy Site Infection	USA	Phage cocktail	Naval Medical Research Center-Frederick	AB	A patient received 8 days of phage therapy with no further signs of infection at the surgical site, though care was ultimately withdrawn due to persistent unresponsiveness.
90	Lerdsittikul et al.	2022	A novel virulent Litunavirus phage possesses therapeutic value against multidrug resistant Pseudomonas aeruginosa	Thailand	vB_PaeS_VL1 (VL1)	sewage	PA	Phage VL1 demonstrated high specificity and efficiency in reducing bacterial load and biofilm formation, significantly increasing the

								survival rate of infected larvae.
91	Leshkasheli et al.	2019	Efficacy of newly isolated and highly potent bacteriophages in a mouse model of extensively drug-resistant Acinetobacter baumannii bacteraemia	Georgia	vB_AbaM_3054 and vB_AbaM_3090	sewage	AB	Phage-based treatments demonstrated high efficacy, achieving approximately 100% survival in both larvae and mice compared to 0% in untreated controls.
92	Li et al.	2024	In vivo efficacy of phage cocktails against carbapenem resistance Acinetobacter baumannii in the rat pneumonia model	China	vB_AbaM_P1 and vB_AbaM_DP45	wastewater	AB	Transcriptomic studies revealed that phage cocktail treatment for pneumonia effectively validated therapeutic efficacy and induced crucial changes in the immune system.
93	Li et al.	2021	A novel broad host range phage phiA85 displays a synergistic effect with antibiotics targeting carbapenem-resistant Klebsiella pneumoniae	China	phiA85	sewage water collected from Capital Institute of Pediatrics,	KP	The combination of phage phiA85 and sublethal antibiotics demonstrated synergistic effects, improving bactericidal

						Beijing, China		efficacy, reducing biofilms, and alleviating pneumonia in mice.
94	Liang et al.	2023	BL02, a phage against carbapenem- and polymyxin-B resistant Klebsiella pneumoniae, isolated from sewage: A preclinical study	China	BL02	sewage	KP	Phage BL02 exhibited high lytic activity and stability, and its single-dose administration resulted in a significantly higher survival rate in infected mice compared to antibiotic treatments.
95	Lin et al.	2025	Isolation and identification of a newly discovered broad-spectrum Acinetobacter baumannii phage and therapeutic validation against pan-resistant Acinetobacter baumannii	China	P425	Medical wastewater	AB	Phage P425 demonstrated potent inhibitory activity and synergistic effects with antibiotics, achieving 100% protection against mortality in mouse infection models when co-administered with levofloxacin.

96	Luo et al.	2024	Synergy of lytic phage pB23 and meropenem combination against carbapenem-resistant Acinetobacter baumannii	China	pB23	sewage	AB	The combination of phage pB23 and meropenem exhibited synergistic antibacterial and antibiofilm effects against carbapenem-resistant A. baumannii in both in vitro and in vivo models.
97	Luo et al.	2022	Bactericidal Synergism between Phage YC#06 and Antibiotics: a Combination Strategy to Target Multidrug-Resistant Acinetobacter baumannii In Vitro and In Vivo	China	phage YC#06	Sewage	AB	Phage-antibiotic synergy (PAS) effectively reduced the required antibiotic concentration, inhibited biofilms, and successfully treated infections in a zebrafish model without inducing phage resistance.
98	Luo et al.	2023	Synergistic Antibacterial Effect of Phage pB3074 in Combination with Antibiotics Targeting Cell Wall against Multidrug-Resistant	China	pB3074	sewage	AB	The combination of phage pB3074 with cefotaxime or meropenem effectively removed mature

			Acinetobacter baumannii In Vitro and Ex Vivo					biofilms and treated wound infections, highlighting its potential as a new antibacterial therapy.
99	Ma et al.	2025	vB_PaeP_PZH3, a novel bacteriophage for the treatment of MDR Pseudomonas aeruginosa in a mouse wound infection model	China	vB_PaeP_PZH3	Hopsital wastewater	PA	Phage vB_PaeP_PZH3 demonstrated strong stability, high antibacterial activity, and significant biofilm-cleaning ability, effectively promoting wound healing in a mouse model.
100	Maan et al.	2025	Efficacy of bacteriophages with Aloe vera extract in formulated cosmetics to combat multidrug-resistant bacteria in skin diseases	Egypt	Nine isolated phages	sewage	PA	A cosmetic gel formulation combining Aloe vera extract and a phage cocktail significantly enhanced phage longevity and reduced bacterial growth by 95.5%.
101	Mani et al.	2025	Augmenting phage therapy using green nanotechnology for promising infection control,	India	M12PA	sewage	PA	The combination of silver nanoparticles and

			wound healing and devoiding phage resistance in MDR Pseudomonas aeruginosa					phages delayed resistance, enhanced antibacterial and antibiofilm efficiency, and demonstrated excellent biocompatibility and wound healing efficacy.
102	Manohar et al.	2018	The therapeutic potential of bacteriophages targeting gram-negative bacteria using Galleria mellonella infection model	India	Klebsiella phage KPP235 (KPP235)	sewage	KP	Multiple doses of a phage cocktail were required to achieve a 100% survival rate in larvae infected with E. coli and E. cloacae, whereas a single dose was sufficient for K. pneumoniae.
103	Martin et al.	2023	Lytic Bacteriophage Is a Promising Adjunct to Common Antibiotics across Cystic Fibrosis Clinical Strains and Culture Models of Pseudomonas aeruginosa Infection	UK	AB-PA01 is a combination of four lytic bacteriophages, Pa193 and Pa204 belonging to the Myoviridae family, and Pa222 and Pa223 belonging to the Podoviridae family	AmpliPhi Australia Pty Ltd.	PA	The combination of a 4-phage cocktail with ceftazidime and tobramycin showed varying synergistic and antagonistic effects on biofilm reduction across

								different clinical strains.
10<br>4	Martins et al.	202<br>2	Effective phage cocktail to combat the rising incidence of extensively drug-resistant Klebsiella pneumoniae sequence type 16	UK	. A phage-cocktail (Katrice-16)	sewage	KP	Phage Katrice-16 demonstrated high in vitro activity, significant anti-biofilm effects, synergistic activity with meropenem, and excellent in vivo rescue capabilities in G. mellonella.
10<br>5	Menon et al.	202<br>1	A Novel N4-Like Bacteriophage Isolated from a Wastewater Source in South India with Activity against Several Multidrug-Resistant Clinical Pseudomonas aeruginosa Isolates	India	vB_Pae_AM.P2 (AM.P2)	wastewater	PA	Phage AM.P2 efficiently lysed P. aeruginosa, showed synergistic bactericidal activity with ciprofloxacin, and successfully inhibited the growth of nearly 30% of MDR clinical isolates.
10<br>6	Michodigni et al.	202<br>2	Formulation of phage cocktails and evaluation of their interaction with antibiotics in inhibiting carbapenemase-producing Klebsiella pneumoniae in vitro in Kenya	Kenya	Cocktail	wastewater	KP	Adjunctive therapy combining imipenem with a two-phage cocktail resulted in a significantly

								lower bacterial culture absorbance compared to the phage cocktail alone.
107	Mohamed et al.	2025	Efficacy of phage vB_Ps_ZCPS13 in controlling Pan-drug-resistant Pseudomonas aeruginosa from urinary tract infections (UTIs) and eradicating biofilms from urinary catheters	Egypt	vB_Ps_ZCPS13	sewage	PA	Phage vB_Ps_ZCPS13 exhibited a wide host range, high stability, significant antibacterial and antibiofilm activity, and showed no cytotoxicity against normal human skin cells.
108	Mohamed et al.	2022	Isolation and Characterization of Bacteriophages Active against Pseudomonas aeruginosa Strains Isolated from Diabetic Foot Infections	Egypt	Two bacteriophages (ϕPAE1 and ϕPAE2)	sewage	PA	Two isolated phages, ϕPAE1 and ϕPAE2, demonstrated a broad host range against P. aeruginosa, maintaining stability across varying temperatures, pH levels, and storage conditions.

109	Mohammadi et al.	2023	Isolation, characterization, therapeutic potency, and genomic analysis of a novel bacteriophage vB_KshKPC-M against carbapenemase-producing Klebsiella pneumoniae strains (CRKP) isolated from Ventilator-associated pneumoniae (VAP) infection of COVID-19 patients	Iran	vB_KshKPC-M	wastewater	KP	Phage vB_KshKPC-M is a stable, lytic phage with a broad host range and high burst size, lacking any known virulence or antibiotic resistance genes.
110	Morris et al.	2025	Phage-antibiotic synergy to combat multidrug resistant strains of Gram-negative ESKAPE pathogens	South Africa	KPW17 targeting K. pneumoniae, clustered with genus Webervirus, ECSR5 targeting E. cloacae clustered with Eclunavirus, PAW33 targeting P. aeruginosa clustered with Bruynoghevirus, while ABTW1 targeting A. baumannii clustered with Vieuvirus.	wastewater	PA, KP, AB	Phage-antibiotic synergy was observed with specific combinations, such as ciprofloxacin with PAW33 and doripenem with KPW17, resulting in the synergistic eradication of tested strains.
111	Mukhopadhyay et al.	2023	Sequential treatment effects on phage-antibiotic synergistic application against multi-drug-resistant Acinetobacter baumannii	China	vB_AbaM-IME-AB2	Beijing Institute of Microbiology and Epidemiology	AB	Simultaneous application of phages and antibiotics demonstrated superior

								antibacterial and antibiofilm activities while effectively suppressing the development of phage resistance.
112	Mulani et al.	2022	Characterization of Novel Klebsiella Phage PG14 and Its Antibiofilm Efficacy	India	PG14	River	KP	Phage PG14 exhibited high stability, a short latent period, and significant inhibition of K. pneumoniae biofilms, nominating it as a potential candidate for phage therapy.
113	Munteanu et al.	2025	Phage Therapy for Orthopaedic Infections: The First Three Cases from the United Kingdom	UK	‘Scrapmetal’ (SCM).	wastewater	KP	The second patient's infection remained unresolved despite receiving phages active against Klebsiella pneumoniae and S. aureus.
114	Nawaz et al.	2025	Characterization of bacteriophages PAA and PAM and evaluation of their antibiotic synergy against Pseudomonas	Pakistan	PAA and PAM	wastewater	PA	Phages PAA and PAM demonstrated stability and synergistic effects

			aeruginosa PAZMYU isolated from urine sample					against planktonic cells and biofilms when combined with sub-inhibitory concentrations of cefepime and meropenem.
115	Ndiaye et al.	2025	Isolation and characterization of Acinetobacter phage vAbaIN10 active against carbapenem-resistant Acinetobacter baumannii (CRAB) isolates from healthcare-associated infections in Dakar, Senegal	senegal	vAbaIN10	wastewater	AB	Phage vAbaIN10 is a highly stable siphovirus with a large burst size that significantly reduced host bacterial growth and lacks any known virulence or resistance genes.
116	Oliveira et al.	2021	Bacteriophage Cocktail-Mediated Inhibition of Pseudomonas aeruginosa Biofilm on Endotracheal Tube Surface	Brazil	A phage cocktail containing 4 × 107 (PFU/mL) of vB_PaeM_USP_2 and vB_PaeM_USP_18	Not mentioned	PA	Phage cocktail coatings on endotracheal tubes significantly reduced biofilms, particularly in multidrug-resistant strains, demonstrating potential for controlling tube-associated infections.

117	Orozco-Ochoa et al.	2025	Bacteriophage Indie resensitizes multidrug-resistant Acinetobacter baumannii to antibiotics in vitro	Mexico	vB_AbaP_Indie	wastewater	AB	The combination of phage Indie and ceftazidime achieved superior bactericidal effects and overcame phage resistance, while its combination with piperacillin-tazobactam showed an antagonistic effect.
118	Pacios et al.	2021	Enhanced Antibacterial Activity of Repurposed Mitomycin C and Imipenem in Combination with the Lytic Phage vB_KpnM-VAC13 against Clinical Isolates of Klebsiella pneumoniae	Spain	vB_KpnM-VAC13	sewage	KP	The combination of lytic phage vB_KpnM-VAC13 with mitomycin C and imipenem successfully killed persister K. pneumoniae isolates and decreased the emergence of resistant mutants.
119	Pallavali et al.	2017	Isolation and in vitro evaluation of bacteriophages against MDR-bacterial isolates from septic wound infections	India	PA DP4 and KP DP1	sewage	KP, PA	Sewage-derived bacteriophages demonstrated perfect lytic activity against MDR bacteria in vitro, suggesting

								their promise as a prophylactic treatment for wound sepsis without enhancing resistance.
120	Paranos et al.	2025	Designing an effective phage cocktail against Klebsiella pneumoniae covering metallo-β-lactamases producing multi-drug resistant clinical isolates	Greece	29 phages were isolated based on plaques' morphology from different pools of tested samples	wastewater	KP	A 5-phage cocktail demonstrated potent lytic activity and rapid distribution in organs, proving highly effective against a wide range of carbapenem-resistant K. pneumoniae isolates.
121	Paranos et al.	2025	Therapeutic application of a jumbo bacteriophage against metallo-β-lactamase producing Pseudomonas aeruginosa clinical isolates	Greece	vB_PaerM_AttikonH10 (AttikonH10)	wastewater	PA	A high dose of the jumbo phage combined with amikacin achieved a significantly greater reduction in bacterial load compared to either monotherapy in a mouse model.
122	Paranos et al.	2025	In Vitro Interactions Between Bacteriophages and Antibacterial Agents of Various	Greece	Five distinct phages (vB_PaerM_AttikonH2, vB_PaerP_AttikonH4,	wastewater	PA	Phages vB_PaerM_AttikonH10 and

			Classes Against Multidrug-Resistant Metallo-β-Lactamase-Producing Pseudomonas aeruginosa Clinical Isolates		vB_PaerM_AttikonH5, vB_PaerM_AttikonH7, and vB_PaerM_AttikonH10)			vB_PaerP_AttikonH4 showed synergistic or additive effects with most tested antibiotics, reversing phenotypic resistance for the majority of strains.
123	Patel et al.	2021	Evaluation of bacteriophage cocktail on septicaemia caused by colistin-resistant Acinetobacter baumannii in immunocompromised mice model	India	ϕAb4, ϕAb7 and ϕAb14	river, ponds and sewer	AB	Simultaneous or prophylactic administration of a phage cocktail prevented mortality, whereas delayed administration resulted in 20 to 60 percent mortality in the bacterial challenge.
124	Peng et al.	2020	Isolation and Characterization of a Novel Phage for Controlling Multidrug-Resistant Klebsiella pneumoniae	China	vB_KleS-HSE3	hospital sewage	KP	Phage vB_KleS-HSE3, a novel Siphoviridae lineage member, demonstrated high antibacterial activity and physical stability against multidrug-

								resistant K. pneumoniae.
125	Pinto et al.	2021	Pseudomonas aeruginosa PAO 1 In Vitro Time–Kill Kinetics Using Single Phages and Phage Formulations—Modulating Death, Adaptation, and Resistance	Portugal	SPCB and SPCG, SMS12, SMS21, and SMS29	sewage and Russian Microgen Sextaphage formulation	PA	A formulation combining all five characterized phages yielded the best time-kill outcomes and reduced resistant variants, though it prompted significant alterations in cell motility and virulence.
126	Pu et al.	2022	Genomic characterization of a new phage BUCT541 against Klebsiella pneumoniae K1-ST23 and efficacy assessment in mouse and Galleria mellonella larvae	China	BUCT541	hospital sewer system	KP	Phage BUCT541 exhibited excellent stability, a wide lysis range, and significantly increased the survival rate and bacterial clearance in in vivo models of MDR-KP ST23 infection.
127	Qadri et al.	2021	Isolation and Identification of a Wastewater Siphoviridae Bacteriophage Targeting Multidrug-resistant Klebsiella pneumoniae	Saudi Arabia	one lytic phage	wastewater	KP	A K. pneumoniae phage isolated from wastewater demonstrated a short latent period, high burst size,

								and stability across various temperatures and pH levels.
128	Quispe-Villegas et al.	2025	In vivo evaluation of phage therapy against Klebsiella pneumoniae using the Galleria mellonella model and molecular characterization of a novel Drulisvirus phage species	Peru	GA23	wastewater	KP	A novel Drulisvirus phage demonstrated effective lytic activity, stability, and improved larval survival, making it a promising candidate for treating MDR K. pneumoniae infections.
129	Qurat-ul-Ain et al.	2021	Efficacy of Phage-Antibiotic Combinations Against Multidrug-Resistant Klebsiella pneumoniae Clinical Isolates	Pakistan	4 isolated phages	hospital sewage	KP	The combination of phages with cefepime and tetracycline displayed promising therapeutic effects by significantly restricting the growth of K. pneumoniae isolates.
130	Racenis et al.	2023	Successful Bacteriophage-Antibiotic Combination Therapy against Multidrug-	Latvia	PNM and PT07	Phages were produced and	PA	A combination of phages, antibiotics, and

			Resistant Pseudomonas aeruginosa Left Ventricular Assist Device Driveline Infection			provided by the Queen Astrid Military Hospital in Brussels, Belgium		surgical intervention successfully cured a relapsing multidrug-resistant P. aeruginosa LVAD driveline infection, preventing biofilm formation.
131	Racenis et al.	2022	Use of Phage Cocktail BFC 1.10 in Combination With Ceftazidime-Avibactam in the Treatment of Multidrug-Resistant Pseudomonas aeruginosa Femur Osteomyelitis-A Case Report	Latvia	The bacteriophage cocktail BFC 1.10	Queen Astrid Military Hospital in Brussels, Belgium	PA	Wound debridement combined with ceftazidime-avibactam and a bacteriophage cocktail successfully eradicated a proximal femoral infection and prevented biofilm formation, though it failed to treat the distal segment.
132	Nsaif Jasim et al.	2018	Formation of therapeutic phage cocktail and endolysin to highly multi-drug resistant Acinetobacter baumannii: in vitro and in vivo study	Iraq	One hundred and thirty six (136) phages specific for 23 A. baumannii bacteria were isolated	Environmental samples	AB	A formulated phage cocktail remarkably minimized bacterial resistance and

								successfully treated all bacteremic mice infected with A. baumannii, while endolysin showed potent antibacterial activity.
133	Rao et al.	2022	Critically Ill Patient with Multidrug-Resistant Acinetobacter baumannii Respiratory Infection Successfully Treated with Intravenous and Nebulized Bacteriophage Therapy	USA	AbW4932 and AbW4878	Phage library	AB	A critically ill patient with a multidrug-resistant A. baumannii respiratory infection was successfully treated using a combination of antibiotics and intravenous and nebulized phage therapy.
134	Rastegar et al.	2024	Characterization of bacteriophage vB_AbaS_SA1 and its synergistic effects with antibiotics against clinical multidrug-resistant Acinetobacter baumannii isolates	Iran	vB_AbaS_SA1	hospital sewage	AB	Phage vB_AbaS_SA1 exhibited a significant phage-antibiotic synergy effect, reducing the overall effective concentration of

								antibiotics required in time-kill assessments.
135	Rastegar et al.	2024	Synergistic effects of bacteriophage cocktail and antibiotics combinations against extensively drug-resistant Acinetobacter baumannii	Iran	SA1, Eve, Ftm, and Gln	hospital sewage	AB	A bacteriophage cocktail effectively inhibited and degraded A. baumannii biofilms, with its efficacy further enhanced when combined with antibiotics.

136	Rezk et al.	2022	Bacteriophage as a potential therapy to control antibiotic-resistant Pseudomonas aeruginosa infection through topical application onto a full-thickness wound in a rat model	Egypt	ZCPA1	Not mentioned	PA	Phage ZCPA1 completely eradicated MDR P. aeruginosa and achieved full wound healing in a rat model, outperforming antibiotics and demonstrating strong potential for topical therapy.
137	Rodríguez-Recio et al.	2025	Genomic Insights into and Lytic Potential of Native Bacteriophages M8-2 and M8-3 Against Clinically Relevant	Mexico	M8-2 and M8-3,	wastewater	PA	Phages M8-2 and M8-3 exhibited specific lytic activity and

			Multidrug-Resistant Pseudomonas aeruginosa					significant efficacy in suppressing MDR P. aeruginosa, positioning them as promising candidates for localized phage therapy.
138	Rotman et al.	2024	Rapid design of bacteriophage cocktails to suppress the burden and virulence of gut-resident carbapenem-resistant Klebsiella pneumoniae	USA	ΦER12 (Ackermannviridae), ΦER15 (Tevenvirinae), ΦER16e (Slopekvirus), ΦER39 (proposed Purpuraviridae), and ΦER46e (Demerecviridae). For UTI-7, we chose ΦMM9 (Autographiviridae), ΦER11 (Tevenvirinae), ΦER27 (Slopekvirus), ΦER32 (Slopekvirus), and ΦER3 (proposed Purpuraviridae).	PhageBank	KP	Optimized phage cocktails selectively suppressed K. pneumoniae in the mouse gut, driving the loss of key virulence factors and charting a roadmap for effective phage therapy.
139	Schooley et al.	2017	Development and Use of Personalized Bacteriophage-Based Therapeutic Cocktails To Treat a Patient with a Disseminated Resistant Acinetobacter baumannii Infection	USA	98 lytic phages	environmental sources by the Biological Defense Research Directorate (BDRD) of	AB	Intravenous and percutaneous administration of bacteriophages successfully cleared an A. baumannii infection and

						the Naval Medical Research Center (NMRC)		reversed the patient's downward clinical trajectory.
140	Senhaji-Kacha et al.	2024	Isolation and characterization of two novel bacteriophages against carbapenem-resistant Klebsiella pneumoniae	Spain	vB_Kpn_F13 and vB_Kpn_F14	hospital sewage	KP	Phages F13 and F14 modestly delayed planktonic bacterial growth.
141	Shafigh Kheljan et al.	2023	Design of Phage-Cocktail–Containing Hydrogel for the Treatment of Pseudomonas aeruginosa–Infected Wounds	Iran	DL52, DL54, DL60, DL62, DL64, and DL68,	wastewater	PA	Phage-antibiotic hydrogels demonstrated a synergistic effect, performing better than antibiotics alone in promoting wound healing and efficiently eliminating P. aeruginosa.
142	Shah et al.	2023	Isolation and characterization of lytic bacteriophage from waste water to control clinical multidrug resistant Klebsiella pneumoniae	Pakistan	RAM-1	Wastewater	KP	Phage RAM-1 demonstrated promising antibiofilm activity, achieving up to a 4-log reduction in K. pneumoniae biofilms after a 6-hour treatment.

143	Shein et al.	2024	Phage cocktail amikacin combination as a potential therapy for bacteremia associated with carbapenemase producing colistin resistant Klebsiella pneumoniae	Thailand	vB_kpnM_05 (myovirus) and vB_kpnP_08 (podovirus)	wastewater	KP	A phage cocktail of vB_kpnM_05 and vB_kpnP_08 provided significant bacteriolysis against XDR K. pneumoniae, though eventual bacterial regrowth suggested the development of phage resistance.
144	Sherif et al.	2025	In vitro, genomic characterization and pre-clinical evaluation of a new thermostable lytic Obolenskvirus phage formulated as a hydrogel against carbapenem-resistant Acinetobacter baumannii	Egypt	VB_AB_Acb75	sewage	AB	A phage-loaded hydrogel significantly improved wound healing and successfully eliminated CRAB infection in a burn-wound animal model compared to the control group.
145	Shi et al.	2021	Safety and Efficacy of a Phage, kpssk3, in an in vivo Model of Carbapenem-Resistant Hypermucoviscous Klebsiella pneumoniae Bacteremia	China	kpssk3	sewage	KP	A single dose of phage kpssk3 protected 100% of mice against bacteremia without causing cytotoxicity or

								notable alterations to the gut microbiota.
146	Shi et al.	2024	Characterization of the novel broad-spectrum lytic phage Phage_Pae01 and its antibiofilm efficacy against Pseudomonas aeruginosa	China	Phage_Pae01	hospital sewage	PA	Phage_Pae01 demonstrated broad-spectrum antibacterial activity, high stability, and effectively disrupted P. aeruginosa biofilms, especially when combined with gentamicin.
147	Shree et al.	2024	Effect of Klebsiella-specific phage on multidrug-resistant Klebsiella pneumoniae- an experimental study	India	Not mentioned	sewage	KP	The combination of phage and imipenem significantly reduced K. pneumoniae colony counts and decreased biofilm production more effectively than phage treatment alone.
148	Singh et al.	2022	Evaluation of Bacteriophage Cocktail on Septicemia Caused by Colistin-	India	ϕKpBHU4, ϕKpBHU7, and ϕKpBHU14	Water sources	KP	A single dose of a phage cocktail protected mice from fatal

			Resistant Klebsiella pneumoniae in Mice Model					septicemia, but higher doses required multiple administrations and were associated with fatal outcomes if given early.
149	Singh et al.	2024	Evaluation of bacteriophage cocktail on urinary tract infection caused by colistin-resistant Klebsiella pneumoniae in mice model	India	ΦKpnBHU1, ΦKpnBHU2 and ΦKpnBHU3	waster sources	KP	Urethral administration of phages effectively eradicated K. pneumoniae, whereas oral and rectal routes required higher concentrations and multiple doses to cure the infection.
150	Sisakhtpour et al.	2022	The characteristic and potential therapeutic effect of isolated multidrug-resistant Acinetobacter baumannii lytic phage	Iran	pIsf-AB02	hospital sewage	AB	Phage pIsf-AB02 rapidly destroyed clinical MDR A. baumannii isolates, exhibited endolysin activity, and safely protected HeLa cells from infection without cytotoxicity.
151	Styles et al.	2022	Investigating Bacteriophages Targeting the Opportunistic	UK	vB_AbaM_PhT2	hospital wastewater	AB	Bacteriophage vB_AbaM_PhT2

			Pathogen Acinetobacter baumannii					demonstrated a 28% host range, lacked virulence factors, protected human cell lines from damage, and showed synergy with colistin.
152	Suchithra et al.	2023	Description and host-range determination of phage PseuPha1, a new species of Pakpunavirus infecting multidrug-resistant clinical strains of Pseudomonas aeruginosa	India	PseuPha1	wastewater	PA	Phage PseuPha1, a new Pakpunavirus species, exhibited strong anti-biofilm activities, high stability, and significant virulence against multi-drug resistant P. aeruginosa.
153	Sun et al.	2025	Characterization of a lytic phage and its efficacy against carbapenem-resistant Pseudomonas aeruginosa infection in mice	China	Pa_WF01	sewage	PA	Phage Pa_WF01 exhibited robust lytic activity, a short latent period, and effectively inhibited bacterial growth across a broad range of pH values and temperatures.
154	Tan et al.	2019	Characterization of Klebsiella pneumoniae ST11 Isolates and	China	117 and phage 31	Not mentioned	KP	A phage cocktail showed significantly

			Their Interactions with Lytic Phages					higher antimicrobial activity than a single phage in broth cultures, highlighting its potential therapeutic value despite rapid resistance development in urine.
155	Tan et al.	2021	Clinical Experience of Personalized Phage Therapy Against Carbapenem-Resistant Acinetobacter baumannii Lung Infection in a Patient With Chronic Obstructive Pulmonary Disease	China	Ab_SZ3	Environmental samples	AB	Personalized nebulized single-phage therapy combined with antibiotics successfully cleared a carbapenem-resistant A. baumannii infection and improved the patient's lung function.
156	Teney et al.	2024	Phage Therapy in a Burn Patient Colonized with Extensively Drug-Resistant Pseudomonas aeruginosa Responsible for Relapsing Ventilator-	France	PP1450, PP1777, PP1792, and PP1797	wastewater	PA	A severely burned patient with extensively drug-resistant P. aeruginosa was successfully

			Associated Pneumonia and Bacteriemia					treated using personalized nebulized and intravenous phage therapy combined with antibiotics and immunostimulation.
157	Thiry et al.	2021	New Bacteriophages against Emerging Lineages ST23 and ST258 of Klebsiella pneumoniae and Efficacy Assessment in Galleria mellonella Larvae	Belgium	hree selected bacteriophages, targeting lineages ST258 (bacteriophages vB_KpnP_KL106-ULIP47 and vB_KpnP_KL106-ULIP54) and ST23 (bacteriophage vB_KpnP_K1-ULIP33)	Wastewater	KP	Treatment with specific podoviruses at a multiplicity of infection of 10 significantly reduced the mortality rate of G. mellonella larvae infected with K. pneumoniae.
158	Tian et al.	2024	Isolation, characterization and therapeutic evaluation of a new Acinetobacter virus Abgy202141 lysing Acinetobacter baumannii	China	Abgy202141	sewage	AB	Acinetobacter virus Abgy202141 demonstrated a short latent period, high stability, and the ability to prevent A. baumannii infections in an in vivo model

								without any virulence genes.
159	Tkhilaishvili et al.	2019	Bacteriophages as Adjuvant to Antibiotics for the Treatment of Periprosthetic Joint Infection Caused by Multidrug-Resistant Pseudomonas aeruginosa	Germany	Not mentioned	collection at the George Eliava Institute of Bacteriophages, Microbiology and Virology	PA	Adjunctive bacteriophage therapy combined with antibiotics successfully eradicated a chronic relapsing multidrug-resistant P. aeruginosa joint and bone infection with no observed side effects.
160	Tsai et al.	2023	Therapeutic effect and anti-biofilm ability assessment of a novel phage, phiPA1-3, against carbapenem-resistant Pseudomonas aeruginosa	Taiwan	phiPA1-3	wastewater	PA	Phage phiPA1-3 is a stable, lytic N4-like Schitoviridae phage that effectively lyses CRPA and shows potential for eradicating P. aeruginosa biofilms.
161	Tunc et al.	2025	Bacteriophage Isolation, Characterization and Antibiofilm Effect Against Multidrug Resistant Gram Negative Bacteria Isolated from Intensive Care Units; Therapeutic Approach	Turkey	MTBB P130581-D, MTBB E830-F, and MTBB K1467-F	wastewater	KP, PA	Phage treatment significantly reduced planktonic bacterial populations and preformed

								biofilms, highlighting its potential as an effective strategy against multidrug-resistant infections.
162	Urgeya et al.	2025	The Ability of Bacteriophages to Reduce Biofilms Produced by Pseudomonas aeruginosa Isolated from Corneal Infections	Australia	DiSu1 to DiSu6	sewage	PA	Phages applied at a higher multiplicity of infection effectively disrupted P. aeruginosa biofilms, though prolonged exposure appeared to promote phage resistance.
163	Van Nieuwenhuyse et al.	2022	Bacteriophage-antibiotic combination therapy against extensively drug-resistant Pseudomonas aeruginosa infection to allow liver transplantation in a toddler	Belgium	two P. aeruginosa phages (PNM and 14-1	Not mentioned	PA	A bacteriophage-antibiotic intravenous combination therapy successfully treated a toddler with extensively drug-resistant P. aeruginosa, allowing for liver retransplantation and complete

								infection resolution.
16 4	Wang et al.	202 1	Colistin-phage combinations decrease antibiotic resistance in Acinetobacter baumannii via changes in envelope architecture	China	Phab24,	river	AB	Phage-resistant A. baumannii mutants exhibited decreased virulence and increased sensitivity to colistin, suggesting a potential clinical advantage of phage-resistance mechanisms.
16 5	Wang et al.	202 1	Phage vB_PaeS-PAJD-1 Rescues Murine Mastitis Infected With Multidrug-Resistant Pseudomonas aeruginosa	China	vB_PaeS_PAJD-1	sewage	PA	Phage PAJD-1 and its endolysin exhibited broad host ranges, strong lytic ability, and effectively reduced bacterial concentrations and repaired mammary glands in mice with mastitis.
16 6	Wang et al.	202 1	Combination Therapy of Phage vB_KpnM_P-KP2 and Gentamicin Combats Acute Pneumonia Caused by K47 Serotype Klebsiella pneumoniae	China	vB_KpnM_P-KP2	sewage	KP	Phage P-KP2 demonstrated high in vitro lysis efficiency and, when combined with gentamicin,

								completely rescued mice from lethal pneumonia caused by K. pneumoniae.
16 7	Wienhold et al.	202 1	Preclinical Assessment of Bacteriophage Therapy against Experimental Acinetobacter baumannii Lung Infection	Germany	vB_AbaM_Acibel004	y Fraunhofer ITEM	AB	Phage treatment significantly reduced pulmonary bacterial burden and inflammation in mice, and its bactericidal effect was confirmed in an ex vivo human lung infection model.
16 8	Wintachai et al.	202 2	Enhanced antibacterial effect of a novel Friunavirus phage vWU2001 in combination with colistin against carbapenem-resistant Acinetobacter baumannii	Thailand	vWU2001	Wastewater	AB	The combination of phage vWU2001 and colistin demonstrated synergistic antimicrobial activity, significantly improving survival and bacterial clearance in G. mellonella compared to either treatment alone.

169	Wintachai et al.	2025	Isolation, Characterization, and Anti-Biofilm Activity of a Novel Kaypoctavirus Against K24 Capsular Type, Multidrug-Resistant Klebsiella pneumoniae Clinical Isolates	Thailand	Phage vB_KpnP_PW7 (vKPPW7)	wastewater	KP	Phage vKPPW7 exhibited high stability, strong lytic activity against K24 capsular type K. pneumoniae, and effectively removed and prevented biofilm formation.
170	Wintachai et al.	2022	Isolation and Characterization of a Phapecoctavirus Infecting Multidrug-Resistant Acinetobacter baumannii in A549 Alveolar Epithelial Cells	Thailand	vABPW7	wastewater	AB	Phage vABPW7 effectively reduced planktonic MDR A. baumannii and biofilm formation on human alveolar epithelial cells without causing cytotoxicity.
171	Wintachai et al.	2022	Isolation and Characterization of a Novel Autographiviridae Phage and Its Combined Effect with Tigecycline in Controlling Multidrug-Resistant Acinetobacter baumannii-Associated Skin and Soft Tissue Infections	Thailand	vB _AbP_ABWU2101	water samples	AB	The combination of phage vABWU2101 and tigecycline demonstrated synergistic antimicrobial and antibiofilm activities, proving more effective

								than either treatment alone.
172	Wintachai et al.	2022	Characterization of Novel Lytic Myoviridae Phage Infecting Multidrug-Resistant Acinetobacter baumannii and Synergistic Antimicrobial Efficacy between Phage and Sacha Inchi Oil	Thailand	vWUPSU	hospital wastewater	AB	The combination of phage vWUPSU and sacha inchi oil significantly inhibited and removed biofilms, demonstrating a synergistic antimicrobial effect against MDR A. baumannii.
173	Xu et al.	2022	The identification of phage vB_1086 of multidrug-resistant Klebsiella pneumoniae and its synergistic effects with ceftriaxone	China	vB_1086	sewage	KP	Phage vB_1086 demonstrated good antibacterial activity when combined with ceftriaxone, and its combination with meropenem effectively inhibited biofilm formation and reduced bacterial resistance.
174	Yakubovskij et al.	2025	Phage vB_KlebPS_265 Active Against Resistant/MDR and Hypermucoid K2 Strains of Klebsiella pneumoniae	Russia	vB_KlebPS_265 (KlebP_265)	sputum	KP	Phage KlebP_265 is a stable, lytic siphophage specific to K2

								strains, whose genome analysis revealed extensive horizontal gene transfer and led to the proposal of new viral subfamilies.
175	Yang et al.	2025	A case report of bacteriophage therapy for the treatment of lung infection due to carbapenem-resistant Pseudomonas aeruginosa	China	Transmission electron microscopy revealed that PaSz-1_45_92k and PaZh_1 belong to the myovirus (Fig. 2A and B), PAL9 belongs to the family of siphovirus (Fig. 2C).	river water	PA	Phage nebulization therapy significantly reduced bacterial load and improved clinical outcomes in a patient, demonstrating that phage resistance can attenuate bacterial virulence.
176	Yin et al.	2017	Phage Abp1 Rescues Human Cells and Mice from Infection by Pan-Drug Resistant Acinetobacter Baumannii	China	Abp1	sewage	AB	Phage Abp1 exhibited high stability, low resistance frequency, and excellent therapeutic efficacy in both local and systemic A. baumannii infection mouse

								models without cytotoxicity.
177	Yoo et al.	2024	Designing phage cocktails to combat the emergence of bacteriophage-resistant mutants in multidrug-resistant Klebsiella pneumonia	SK	four phages (U2874, phi_KPN_H2, phi_KPN_S3, and phi_KPN_HS3)	Environmental samples	KP	Phage cocktails that induce resensitization of phage susceptibility exhibit superior resistance-suppressing ability, highlighting the importance of host range data against resistant mutants.
178	Yuan et al.	2019	Efficacy of a phage cocktail in controlling phage resistance development in multidrug resistant Acinetobacter baumannii	China	vB_AbaS_D0	sewage	AB	A cocktail combining phages vB_AbaS_D0 and vB_AbaP_D2 significantly improved therapeutic efficacy and lowered the frequency of resistance mutations compared to single phage treatments.
179	Zagaliotis et al.	2025	Bacteriophage treatment is effective against carbapenem-	USA	Spivey, Pharr, and Soft	Not mentioned	KP	A combination of bacteriophages

			resistant Klebsiella pneumoniae (KPC) in a neutropenic murine model of gastrointestinal translocation and renal infection					and ceftazidime-avibactam produced a synergistic effect, significantly reducing the bacterial burden in a mouse model of disseminated KPC infection.
180	Zaki et al.	2023	Characterization and comprehensive genome analysis of novel bacteriophage, vB_Kpn_ZCKp20p, with lytic and anti-biofilm potential against clinical multidrug-resistant Klebsiella pneumoniae	Egypt	vB_Kpn_ZCKp20p	urban and medical sewage	KP	Phage vB_Kpn_ZCKp20p demonstrated high lytic efficiency, extended stability, and antibiofilm activity, representing a safe, strictly lytic new species for potential therapy.
181	Zhang et al.	2024	Characterization and therapeutic potential of MRABP9, a novel lytic bacteriophage infecting multidrug-resistant Acinetobacter baumannii clinical strains	China	MRABP9	sewage	AB	Phage MRABP9 showed potent bactericidal and anti-biofilm activity, successfully rescuing mice from acute lethal MRAB infection, making it a

								promising therapeutic candidate.
182	Zhao et al.	2024	Antibacterial effect of phage cocktails and phage-antibiotic synergy against pathogenic Klebsiella pneumoniae	China	total of seven phages were selected, which were distributed in different groups (Fig. 2), and the negative staining electron microscopy images indicate that seven selected phages possess three different morphological phages (Fig. 3a; Fig. S1), phage P52 with a contractile tail, four phages with a short tail (P60, P67, P79, and P85), and two phages containing a long non-contractile tail (P28 and P61). The whole genome sequences revealed that these seven phages belong to Caudoviricetes class (P85 and P61), Slopekvirus genus (P52 and P60), Webervirus genus (P28), and Przondovirus genus (P67 and P79).	wastewater	KP	Optimized phage-antibiotic synergy demonstrated broad-spectrum antibacterial potential and significantly reduced bacterial burden in a murine model by suppressing pathogen density and resistance.

183	Zheng et al.	2024	Antibacterial activity evaluation of a novel K3-specific phage against Acinetobacter baumannii and evidence for receptor-binding domain transfer across morphologies	China	P1068	wastewater	AB	Phage P1068 demonstrated strong antimicrobial activity against A. baumannii and significantly reduced bacterial loads in infected mice, while revealing potential horizontal transfer of tail fiber genes.
184	Zhu et al.	2025	In vitro and in vivo antibacterial efficacy of bacteriophage combined with tigecycline against carbapenem-resistant Klebsiella pneumoniae and characterization of phage resistant mutants	China	HZJ31	hospital wastewater	KP	Phage HZJ31 exhibited remarkable anti-biofilm activity and synergistically improved survival rates when combined with tigecycline, while phage-resistant mutants showed reduced virulence.
185	Ziller et al.	2024	Newly isolated Drexlerviridae phage LAPAZ is physically robust and fosters eradication of Klebsiella pneumoniae in combination with meropenem	Germany	LAPAZ, vB_KpnD-LAPAZ	hospital wastewater	KP	Phage LAPAZ achieved complete bacterial eradication and delayed resistance when combined with meropenem,

								while its resistant mutants exhibited altered drug sensitivities due to a specific gene mutation.

KP: Klebsiella pneumoniae

AB: Acinetobacter baumannii

PA: Pseudomonas aeruginosa
